# Nature-inspired stochastic hybrid technique for joint and individual inversion of DC and MT data

**DOI:** 10.1038/s41598-023-29040-x

**Published:** 2023-02-15

**Authors:** Kuldeep Sarkar, Mukesh, Upendra K. Singh

**Affiliations:** Department of Applied Geophysics, IIT(ISM), Dhanbad, 826004 India

**Keywords:** Solid Earth sciences, Geophysics, Hydrogeology

## Abstract

Here, a new naturally-inspired stochastic nonlinear joint and individual inversion technique for integrating direct current (DC) and magnetotelluric (MT) data interpretation-based simulation of a swarm intelligence combo with specific capabilities for exploitation of the variable weight particle swarm optimizer (vPSO) and exploration of the grey wolf optimizer (GWO), vPSOGWO, is used. They are particularly notable for their capacity for information exchange while hunting for food. Through synthetic MT and DC data contaminated with various sets of random noise, the applicability of the anticipated vPSOGWO algorithm based joint and individual inversion algorithm was assessed. The field examples, collected from diversified different geological terrains, including Digha (West Bengal), India; Sundar Pahari (Jharkhand), India; Puga Valley (Ladakh), India; New Brunswick, Canada; and South Central Australia, have shown the practical application of the proposed algorithm. Further, a Bayesian probability density function (bpdf) for estimating a mean global model and uncertainty assessment in posterior; and a histogram for model resolution assessment have also been created using 1000 inverted models. We examined the inverted outcomes and compared them with results from other cutting-edge methodologies, including the GWO, PSO, genetic algorithm (GA), Levenberg–Marquardt (LM), and ridge-regression (RR). Our findings showed that the current methodology is more effective than the GWO, PSO, GA, LM, and RR techniques at consistently improving the convergence of the global minimum. In contrast to earlier approaches, the current cutting-edge strategy vPSOGWO offers an improved resolution of an additional significant crustal thickness of about 65.68 ± 1.96 km over the Puga Valley, in which the inverted crustal thickness determined by vPSOGWO agrees well with the published crustal thickness over the Puga Valley. The new technology brings simulations closer to genuine models by significantly reducing uncertainty and enhancing model resolution.

## Introduction

The evolution of geophysical techniques over the last several decades has made them an essential instrument for addressing a variety of geological issues as well as for locating groundwater, mineral, hydrocarbon reservoir, and near-subsurface research^1–5^. Since model parameters and geophysical datasets are not linearly related, it has become challenging to deduce this from geophysical data. As a result, inversion techniques are needed to interpret geophysical data, which are employed by both local and global optimization techniques^6–9^. Most frequently, local optimization techniques (e.g., Conjugate gradient, Levenberg–Marquardt/Ridge-Regression, Newton-Gauss, Steepest descent, Occam, etc.) are used to identify the global solution, which needs a starting model that is somewhat similar to the real model to achieve successful convergence. Nevertheless, this technique frequently traps users at local minima, leading to models with built-in non-uniqueness^10,11^. On the other hand, global inversion procedures require a broad search space for model parameters to prevent convergence to local minima, which works well when the starting model is flawed in local Optimization. This Optimization begins with random models to identify optimum solutions without calculating the Jacobian matrix or derivatives.

Due to technological development, a variety of global inversions-based metaheuristic algorithms (swarm intelligence) such as Ant colony (ACO), Bat algorithm (BA), Particle Swarm (PSO), Grey Wolf (GWO), Firefly algorithm, etc.,^12–16^, an evolutionary algorithm such as Genetic Algorithm (GA), Differential Evolution (DE)^17,18^ and physics and chemistry-based algorithms namely Gravitation Search Algorithms (GSA), big-bang-big-crunch, simulating annealing, SA^19–21^ over geophysical data has been increased since the beginning of the twenty-first century, where no initial guesses require but needs search range to find out the reliable solution and avoid entrapment in local minima owing to its flexibility and stochastic character^22^.

Swarm intelligence (SI), as stated by Mirjalili et al.^16^, has fewer tuning parameters and operators and is easier to save the best model at each iteration than other algorithms. However, to get a better convergent solution, optimization techniques need exploration and exploitation capabilities that stand in balance with one another, i.e., as one capability increases, the other decreases, and vice versa^23^. SI is preferred over all others since it can preserve information over iterations with fewer tuning adjustments^16^. As a result, integrating two metaheuristic algorithms with opposing capabilities involves several researchers. Thus, the PSO algorithm is frequently hybridized due to its high potential for use, rapid convergence, and simplicity. For example includes PSO with GA, PSOGA^24,25^, PSO with DE, PSODE^26^, PSO with ACO, PSOACO^27^, and PSO with GSA, PSOGSA^28^, PSO with GWO, PSOGWO^23^, etc.

To solve the geophysical inverse problems, a joint inversion and individual inversion of Direct Current (DC) and Magnetotelluric (MT) data using a new vPSOGWO approach are promoted here due to its better ability to converge the algorithm and search the global model with the least uncertainty^29,30^. The vPSOGWO algorithm is initially demonstrated using various sets of simulated synthetic data with a 10% noise setting. This inversion technique utilized DC and MT field datasets from different geological contexts based on how well these algorithms performed when employed with synthetic datasets. The Bayesian posterior probability density function (bpdf) is calculated using those inverted models with a 68.27% confidence interval to determine the mean model and access the posterior uncertainty. A correlation matrix is also estimated to assess the relationship between layer parameters. We evaluated the effectiveness of the inverted results and compared them by examining their uncertainty, stability, sensitivity, and resolution. We discovered that the vPSOGWO produces reliable, comparable, and more precise with lower posterior uncertainty than results inverted by other techniques.

Following is how this study is organized: (i) a novel stochastic metaheuristic optimization techniques are derived and designed with various kinds of MT and DC synthetic examples distorted with 10% Gaussian noise to show how the new strategy can reliably estimate each model parameter, test the sensitivity and novelty of the new technology, as described in the “Synthetic examples” Section, (ii) the traditional deterministic gradient methods and some stochastic inversion techniques are reviewed, (iii) the Bayesian probability density function and covariance matrix are formulated and analyzed to reduce the uncertainty in posterior inverted results and ensure the uniqueness of the solution as described in "Results and discussion" Section. It is shown how the present algorithm, vPSOGWO, with the better performance achieved, when they were used to predict the earth's subsurface structure across different geological setups as discussed in “Field examples” Section and (iv) The benefits and drawbacks of the new methodology are covered in the final part in “Conclusion” Section, where we also make some recommendations for potential future research.

## Method

### Forward modeling formulation

Taking into account that the 1D depth model of the Earth's subsurface consists of (*p*-1) number of subsurface interfaces and *p* number of subsurface layers. In this scenario, the associated electrical resistivity of the subsurface layer will be $\rho = \left[ { \rho_{1} , \rho_{2} , \ldots \rho_{p - 1} , \rho_{p} } \right]$, and the thickness between consecutive interfaces will be $h$ = [$h_{1}$, $h_{2} , \ldots h_{p - 1}$]. For a semi-infinite half-space, the final layer's thickness is considered infinite. In a joint inversion,

$$\vec{x} = \{ \rho_{1} , \rho_{2} , \ldots \rho_{p - 1} , \rho_{p} , h_{1} ,h_{2} , \ldots h_{p - 1} \} ,$$

Consequently, the sum of all the layer parameters *p* + (*p *− 1). In contrast, many articles and books provide the basic one-dimensional multi-layered forward modeling formulas for Magnetotelluric (MT) electromagnetic and direct current (DC) electrical resistivity data, and a few related expressions used in the study are presented below.

#### DC resistivity sounding method

The apparent resistivity (${\rho }_{aDC}$) for a multi-layered earth surface for the Schlumberger sounding array at half the current electrode spacing (*s*) is as follows^31,32^:

1 $$\rho_{aDC} \left( {s,x} \right) = s^{2} \mathop \smallint \limits_{0}^{\infty } \tau \left( \lambda \right)\lambda J_{1} \left( {\lambda s} \right)d\lambda$$

where ${J}_{1}$ and $\tau (\lambda )$ are the first-order Bessel function and the resistivity transform.

The resistivity transforms for the first layer of (*p *− 1) are given by:

2 $$\tau_{p - 1} \left( \lambda \right) = \frac{{\tau_{p} \left( \lambda \right) + \rho_{p - 1} \tanh \left( {\lambda h_{p - 1} } \right)}}{{1 + \tau_{p} \left( \lambda \right)\tanh \left( {\lambda h_{p - 1} } \right)/\rho_{p - 1} }}$$

The resistivity transforms for the $p$th layer is ${\tau }_{p}\left(\lambda \right)={\rho }_{p}$, which denotes that the resistivity transforms for the $p$th layer is equal to the resistivity of the half-real space by ${h}_{p}$ and ${\rho }_{p}$, respectively, reflecting the $p$th layer's thickness and resistivity.

#### MT sounding method

The impedance Z for a multi-layered earth surface is described as a function of frequency that provides the impedance for the first to ($p - 1$)th layer as follows^33^:

3 $$Z_{p} \left( \omega \right) = \frac{{Z_{p + 1} + T_{p} }}{{1 + S_{p} Z_{p + 1} }}$$

The resistivity transforms for $p$-layered systems is ${Z}_{p}=\omega \sqrt{{\rho }_{p}}$, implying that the impedance of the $p$th layer is proportional to the real resistivity of the half-space.

Here,

4 $$T_{p} = \omega \sqrt {\rho_{p} } \tanh \left( {\frac{{\omega h_{p} }}{{\sqrt {\rho_{p} } }}} \right)$$

5 $$S_{p} = \frac{1}{{\omega \sqrt {\rho_{p} } }}\tanh \left( {\frac{{\omega h_{p} }}{{\sqrt {\rho_{p} } }}} \right)$$

Hence, the apparent resistivity (${\rho }_{aMT}$) for magnetotelluric (MT) sounding is defined as:

6 $$\rho_{aMT} = \frac{1}{\mu \omega }Z^{*} Z$$

where Z*, $\mu$, and $\omega$ are the impedance's complex conjugate, the magnetic permeability of the medium, and angular frequency, respectively.

### Inverse modelling

Using a mathematical inverse theory, it is possible to derive the solution or model (x) from the observed datasets (d). Based on the physical laws, the inverse solution is a source for computing the datasets from a given model, known as forward modelling, is defined with forward operator (G) as:

7 $$d = G\left( x \right)$$

To estimate the best model, the cost function is minimized using Eq. (7) as given below:

8 $$Cost\_function = \frac{1}{n}\mathop \sum \limits_{j = 1}^{n} \left( {d\_obs_{j} - d\_cal_{j} } \right)^{2}$$

where *n* is the number of observation points, j represents 1, 2,…, n, $d\_obs$ is the observed apparent resistivity data and $d\_cal$ is the calculated apparent resistivity data.

Here, the noisy synthetic data ($${d}_{noise})$$ with synthetic data ($${d}_{syn})$$ and Gaussian random variable [0, 1] is created using the following:

9 $$d_{noise} = d_{syn} + \left( {2 \times rand - 1} \right)d_{syn} \times Noise\%$$

Our novel algorithm namely vPSOGWO, GWO, and PSO randomly select a starting value from the given search space. In this situation, the final criterion was taken as the number of iterations.

#### Particle swarm optimizer

**Figure 1:**
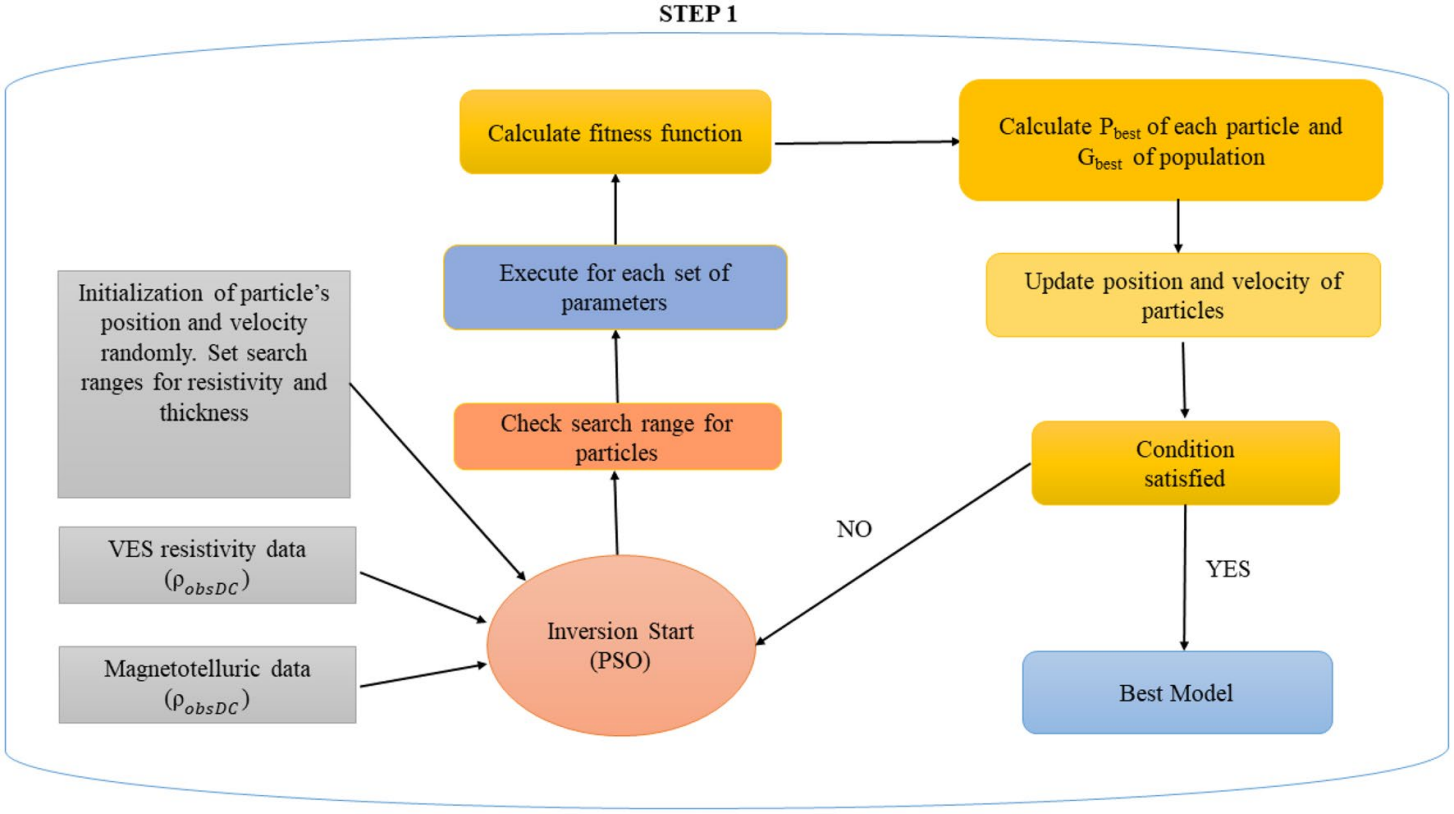
A schematic flow diagram depicts the procedures for individual and joint inversion of DC and MT data-based Particle Swarm Optimization.

Particle Swarm Optimizer (PSO) mimics the natural behavior of particles seeking nourishment using collaborative support from a model population represented by resistivity layer parameters/solutions/models (called particles) in a swarming group^34^. Moreover, PSO was examined to have high exploitation and low exploration ability^23^. For each iteration, the best solution/position acquired among the particles thus far is saved, which aids in the search for the optimal solution, which is determined by the fitness of each particle assessed using Eq. (8). Equations (10) and (11) update or specify the velocity, ${\overrightarrow{v}}_{k}\left(t+1\right)$, and location of the particles, ${\overrightarrow{x}}_{k}\left(t+1\right)$, in the search space for the *k*th particle at the *t*th iteration with inertia weight, w, varies between 0 and 1 while updating the velocity and position, Particles shift their positions with each iteration to find the best answer. In Eq. (10), the first, second, and third terms indicate exploratory ability, private thought, and particle cooperation, respectively^14^.

10 $$\vec{v}_{k} \left( {t + 1} \right) = w\vec{v}_{k} \left( t \right) + c_{1} \times rand \times \left( {\vec{x}_{p} - \vec{x}_{k} \left( t \right)} \right) + c_{2} \times rand \times \left( {\vec{x}_{g} - \vec{x}_{k} \left( t \right)} \right)$$

11 $$\vec{x}_{k} \left( {t + 1} \right) = \vec{x}_{k} \left( t \right) + \vec{v}_{k} \left( {t + 1} \right)$$

where ${c}_{1}$ and ${c}_{2}$ are a personal learning and global learning coefficient, respectively, and *rand* is used for a random number ranging from 0 to 1, ${\overrightarrow{x}}_{p}$ is the current best solution, ${\overrightarrow{x}}_{g}$ is the global best solution, ${\overrightarrow{x}}_{k}\left(t\right)$ is the position of the *k*th particle at *t*th iteration. The schematic flow chart diagram of PSO is illustrated in Fig. 1.

#### Grey wolf optimizer

Grey Wolf Optimizer** (**GWO) algorithm is based on the nature of grey wolves, which mimics the leadership hierarchy and hunting dynamics of grey wolves. It leverages its capacity to solve standard and real-life issues^16^. This GWO algorithm was examined to have high exploration and low exploitation ability^23^. Grey wolves are classified into four groups: alpha, beta, delta, and omega. The alphas are the pack leaders who make significant and final decisions for all the wolves. The betas are subordinates who assist the alphas in making decisions, but they cannot compel or command them; they can only order the lesser wolves. The beta group gets the command from the alpha group and reinforces it throughout the other group before providing feedback to the alpha. All of the groupings dominate the omega wolves. The omega group is an important part of hunting as they play the role of scapegoat and are always allowed to eat at the end.

If a wolf does not belong to the alpha, beta, or omega groups, they are classified as delta, which only summits the alpha and beta groups. The alpha group is the best answer in the GWO algorithm. The beta and delta groups are the best consecutive solutions, while the omega group is the best contender for a solution. The omega group always comes after the others. Prey searching, surrounding the prey, and assaulting the prey are the three types of hunting. The following equation shows the encircling nature of the wolves:

12 $$\vec{d} = \left| {c\left( t \right) - \vec{x}\left( t \right)} \right|$$

13 $$\vec{x}\left( {t + 1} \right) = \vec{x}_{p} \left( t \right) - ad$$

where $\vec{x}_{p}$ is the prey position, $\vec{x}$ is the grey wolf position, $t$ is the iteration, $\vec{a}$ and $\vec{c}$ are the vectors mathematically formulated as:

14 $$\vec{a} = \overrightarrow {a1} \left( {2 \times rand - 1} \right)$$

where $$\overrightarrow {a1} = 2\left( {1 - iter/l} \right)$$.

15 $$\vec{c} = 2 \times rand$$

The values range from [− 2$\overrightarrow{a1}$, 2$\overrightarrow{a1}$], which are used by wolves to induce the search to shift away from the prey.

If $\overrightarrow{a}\ge$ 1, then find a better answer, the hunting is put on hold,

and If $\overrightarrow{a}<$ 1, then the wolves are forced to attack the prey.

Here, the value of a1 ranges from 2 to 0 in decreasing order with increasing iteration (*iter*), and $l$ represents the maximum iteration.

The alpha group dominated the grey wolf colony, with the beta and delta groups searching for prey and the omega groups following them. In the GWO method, the alpha group wolves provide the best answer, while the beta and delta group wolves supply the second and third best solutions, respectively. As a result, the remainder of the community wolves, i.e., the omega group wolves, follow the best solution wolves to acquire a better location, which is described mathematically as:

16 $$\vec{d}_{\alpha ,\beta ,\delta } = \left| {\vec{c}_{1,2,3} \times \vec{x}_{\alpha ,\beta ,\delta } - \vec{x}} \right|$$

$\vec{x}_{\alpha }$, $\vec{x}_{\beta }$, and $\vec{x}_{\delta }$, respectively, indicate the ideal location for alpha, beta, and delta wolves in each iteration.

17 $$\vec{x}_{1,2,3} = \left| {\vec{x}_{\alpha ,\beta ,\delta } - \vec{a}_{1,2,3} \times \vec{d}_{\alpha ,\beta ,\delta } } \right|$$

Here, $\vec{x}_{p} \left( {t + 1} \right)$ describes the updated location of the prey in $\left( {{\text{the}}\;t + 1} \right)$ iteration, which is derived from the mean position of the three best wolves in the population, that is,

18 $$\vec{x}_{p} \left( {t + 1} \right) = \left( {\vec{x}_{1} + \vec{x}_{2} + \vec{x}_{3} } \right)/3$$

**Figure 2:**
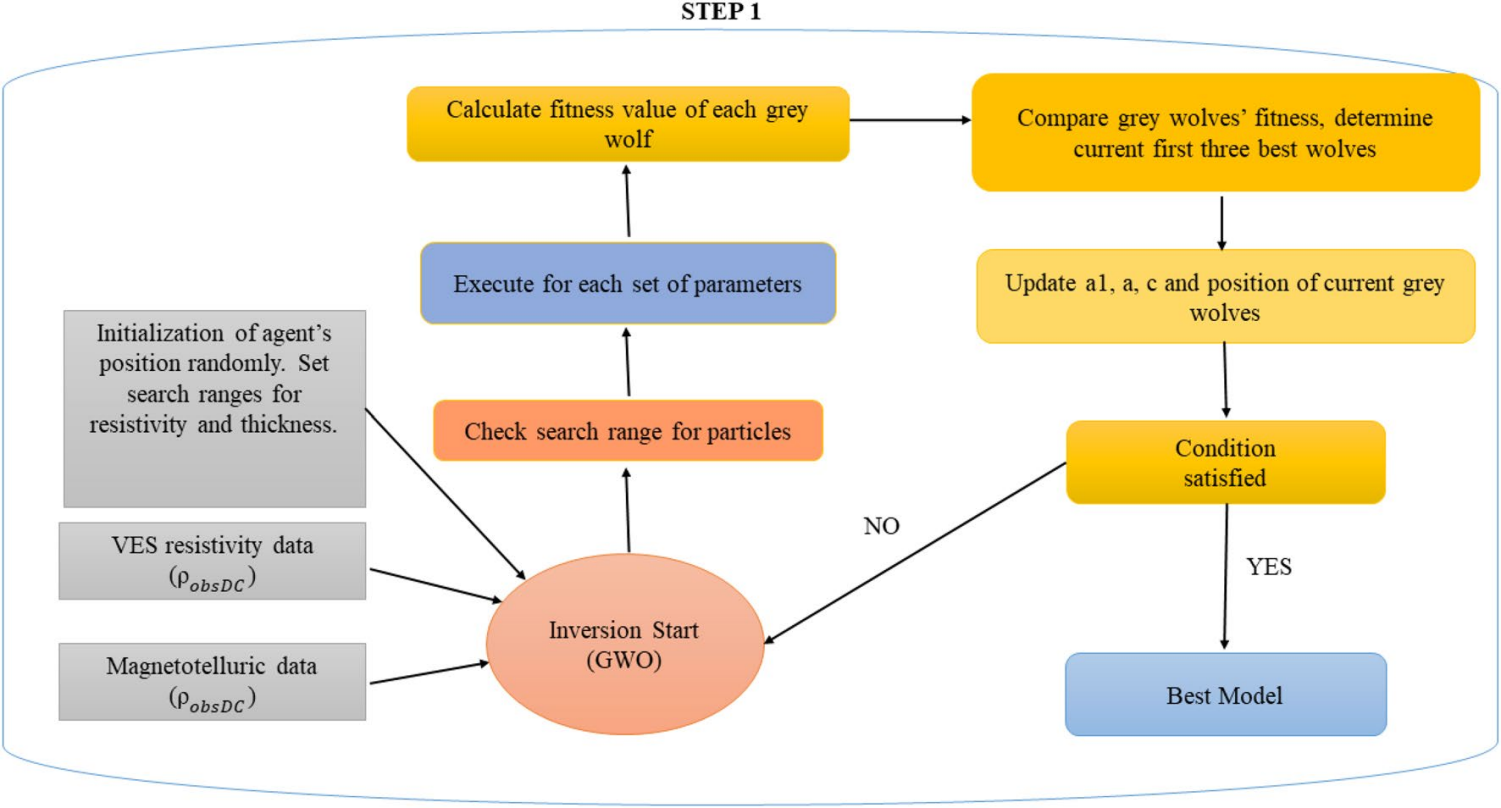
The procedures for individual and joint inversion of DC and MT data-based Grey Wolf Optimization are shown in the schematic flow diagram.

The schematic flow chart diagram of GWO is illustrated in Fig. 2.

#### Variable particle swarm–grey wolf optimizer

Variable Particle Swarm–Grey Wolf Optimizer (vPSOGWO) is a low-level mixed co-evolutionary approach because the functions of both algorithms PSO and GWO are combined to offer the final solution^23^. This hybrid algorithm balances GWO's exploitation and PSO's exploration capabilities. The position of the alpha ($\mathrm{\alpha }$), beta ($\upbeta$), and delta ($\updelta$) wolves of the GWO algorithm that encircle the prey in the vPSOGWO algorithm aid in updating the position of the swarm in the PSO algorithm. Similarly, bird placements assist in updating wolf positions in search space.

The following formulae are used to update the encircling location of wolves:

19 $$\vec{D}_{{{\upalpha },{\upbeta },{\updelta }}} = \left| {Const \times \vec{X}_{{{\upalpha },{\upbeta },{\updelta }}} \left( t \right) - w \times \vec{x}_{k} } \right|$$

20 $$\vec{x}_{1,2,3} = \left| {\vec{X}_{{{\upalpha },{\upbeta },{\updelta }}} \left( t \right) - A_{1,2,3} \times \vec{D}_{{{\upalpha },{\upbeta },{\updelta }}} } \right|$$

where

21 $$\vec{A}_{1,2,3} = \left( {2\vec{a} \times rand - \vec{a}} \right),$$

22 $$\vec{a} = 2 - 2 \times iter/l,$$

Here, $l$ represents the total number of iterations, $Const$ is 0.5, and ${\overrightarrow{x}}_{k}$ denotes the random resistivity model chosen by each particle or agent. ${\overrightarrow{D}}_{\mathrm{\alpha },\upbeta ,\updelta }$ and ${\overrightarrow{X}}_{\mathrm{\alpha },\upbeta ,\updelta }$ are the distance vectors and position/model of alpha (α), beta (β), and delta (δ), respectively; and ${\overrightarrow{x}}_{1,\mathrm{2,3}}$ are the three best-updated positions/models of prey/real model updated by α, β, and δ.

The following equation^29,30,35^ is used in PSO to update the position/model (${\overrightarrow{{\varvec{x}}}}_{k}$) and velocity (${\overrightarrow{{\varvec{v}}}}_{k}$) of the $k$-th particle:

23 $$\begin{gathered} \vec{v}_{k} \left( {t + 1} \right) = w\vec{v}_{k} \left( t \right) + c_{1} \times rand \times \left( {\vec{x}_{1} - \vec{x}_{k} \left( t \right)} \right) + c_{2} \times rand \hfill \\ \quad \quad \quad \quad \quad \; \times \left( {\vec{x}_{2} - \vec{x}_{k} \left( t \right)} \right) + c_{3} \times rand \times \left( {\vec{x}_{3} - \vec{x}_{k} \left( t \right)} \right) \hfill \\ \end{gathered}$$

24 $$\vec{x}_{k} \left( {t + 1} \right) = \vec{x}_{k} \left( t \right) + w\vec{v}_{k} \left( {t + 1} \right)$$

where

25 $$w = w_{max} - \left( {w_{max} - w_{min} } \right)iter/l$$

Here, ${w}_{max}=0.9$, ${w}_{min}=0.2$, and ${\overrightarrow{a}}_{\mathrm{1,2},3}$ are more appropriate after tuning for our study^29^.

**Figure 3:**
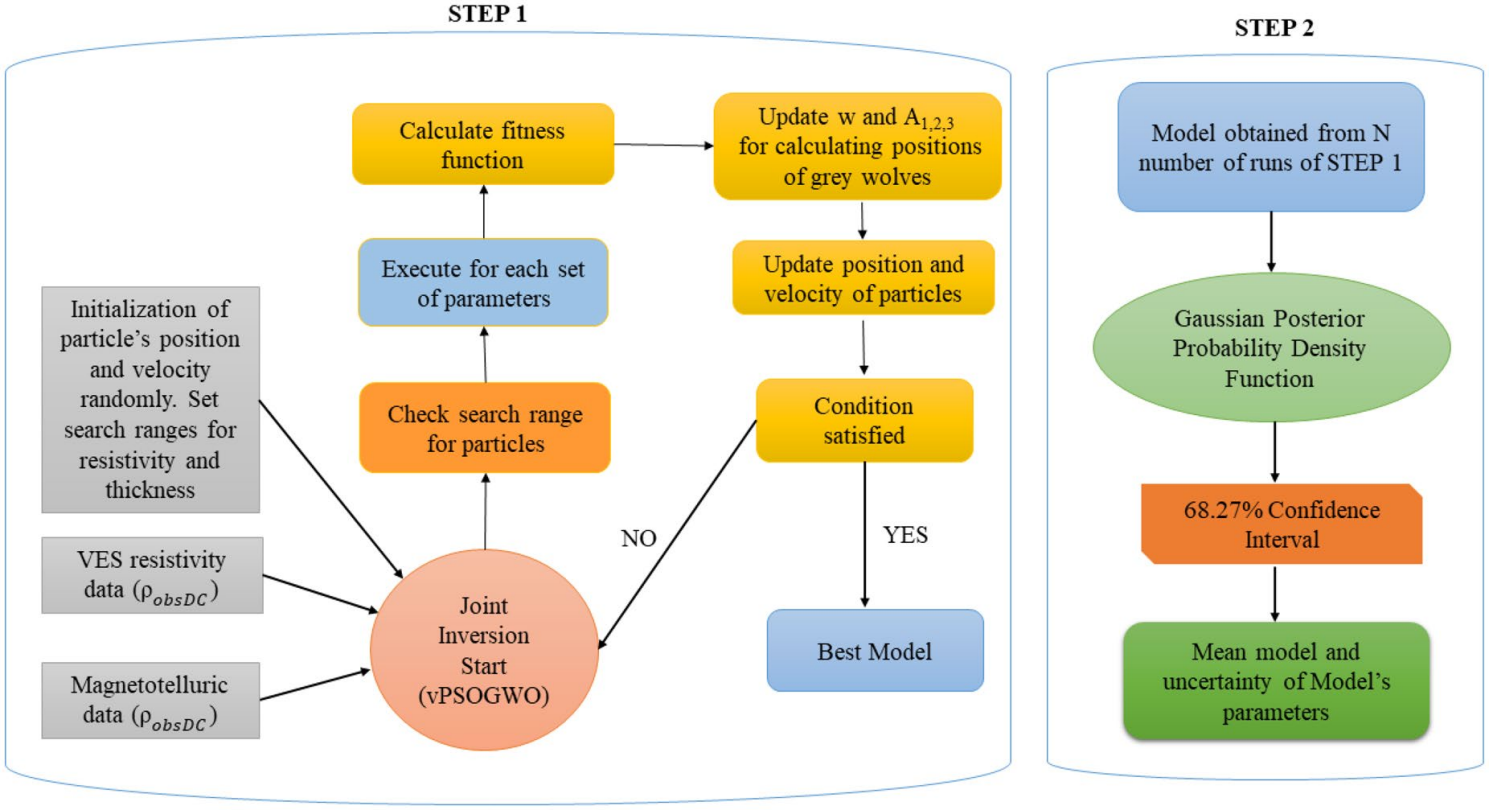
The procedures for individual and joint inversion of DC and MT data-based vPSOGWO are illustrated in a schematic flow diagram.

The schematic flow chart diagram of vPSOGWO is illustrated in Fig. 3.

##### Pseudo-code of vPSOGWO algorithm

*Max_Iter*: maximum iterations (*l*)

*Pop_no*: population size ($z$)

*Para*: Number of parameters ($i$)

*Fitness*: set to infinity

*LB* and *UB*: set Lower bound (*LB*) and Upper bound (*UB*) for different parameters

Initialize particles/resistivity models randomly
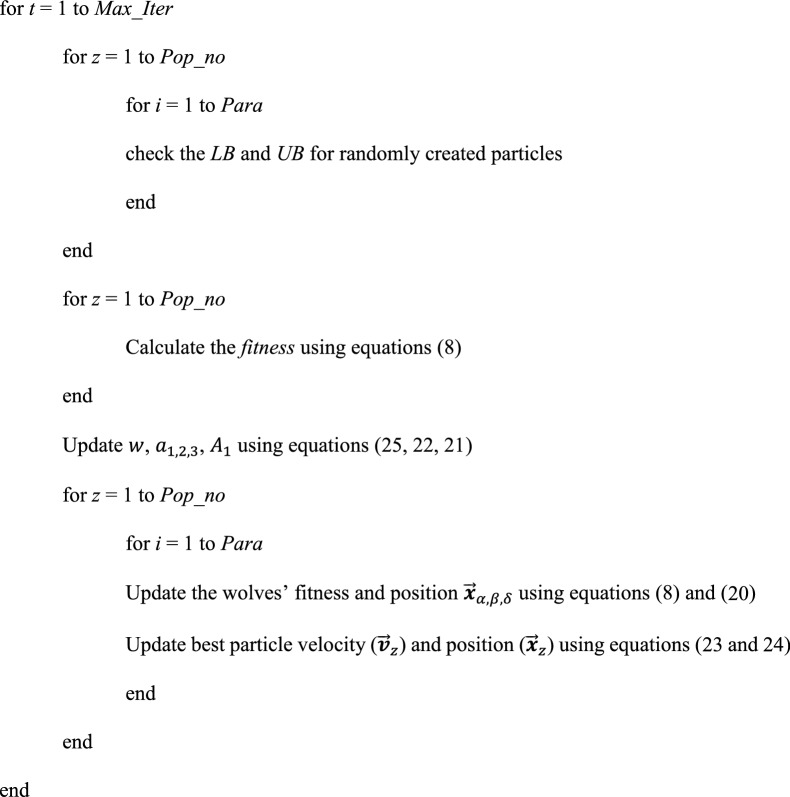


### Bayesian probability density function and confidence interval

The probability Density Function (*pdf*) is a statistics function that explains the probability of random variables within a given range of values. It produces the likelihood of values for random variables. The one-dimensional posterior Bayesian probability density function (*bpdf*) for various parameters ($$i)$$ is defined as^36^:

26 $$P\left( {x_{ij} {|}\rho_{obs} } \right) = \frac{{f\left( {x_{i} } \right)f\left( {\rho_{obs} |x_{j} } \right)}}{{\mathop \sum \nolimits_{j} f\left( {x_{i} } \right)f\left( {\rho_{obs} |x_{j} } \right)}}$$

where $P\left({x}_{ij}|{\rho }_{obs}\right)$ is the posterior Bayesian probability density/distribution function of the parameter (${x}_{ij}$) given the evidence (${\rho }_{obs}$), and $f\left({x}_{i}\right)$ is the priori distribution function for each parameter.

The priori distribution function, $f\left({x}_{i}\right)$, and the likelihood function, $f\left({\rho }_{obs}|{x}_{j}\right)$^36^ are defined as:

27 $$f\left( {x_{i} } \right) = \left\{ \begin{gathered} \frac{1}{{UB_{i} - LB_{i} }},\quad LB_{i} \le x_{i} \le UB_{i} \hfill \\ 0,\quad \quad \quad \quad \quad \;\;elsewhere \hfill \\ \end{gathered} \right.$$

28 $$f\left( {\rho_{obs} |x_{j} } \right) = \mathop \prod \limits_{i = 1}^{n} \frac{1}{{\sqrt {2\pi \sigma^{2} } }} \exp \left\{ { - \frac{{\left( {G(x_{j} ) - \rho_{obs} } \right)^{2} }}{{2\sigma^{2} }}} \right\}$$

where ${\rho }_{obs}$, ${G(x}_{j})$, ${\sigma }^{2}$, and ${\widehat{m}}_{i}$ are the observed apparent resistivity, calculated apparent resistivity, variance, and mean of the distribution resulting from model $x$ for $j$th run, respectively. ${LB}_{i}$ and ${UB}_{i}$ are the lower and upper search range of each parameters.

Consequently, the mean model ($$\widehat{m}$$), and standard deviation ($$\sigma$$) of the model parameters with a total number of models ($$M)$$, are defined as^37^:

29 $$\hat{m}_{i} = \frac{1}{M}\mathop \sum \limits_{j = 1}^{M} x_{i,j}$$

30 $$\sigma_{i} = \sqrt {\frac{1}{M - 1}\mathop \sum \limits_{j = 1}^{M} \left( {x_{i,j} - \hat{m}_{i} } \right)^{2} }$$

For further study, the models derived from several iterations of the inversion process are employed, and posterior pdf and histogram are computed for all acceptable models. The probability distribution of the inverted models is calculated using a posterior Bayesian pdf, and the resolution of the inversion technique is determined using a histogram. The study continues with the confidence interval of parameters, which is the probability that a value of parameter ${m}_{i}$ falls within a specific range of the mean, ${\widehat{m}}_{i}$^38^. As a result, the confidence interval (CI) is directly proportional to the area distribution in posterior bpdf ($P$). According to the empirical rule stated by Ross^37^, 68.27% of the inverted models are within one standard deviation. Thus, the model parameters obtained from vPSOGWO within the bpdf (> 68.27% CI) are accepted for calculating the mean solution and uncertainty, giving the model a near approximation to the global solution with reduced uncertainty.

## Results and discussion

The applicability of the developed new algorithm, namely vPSOGWO for individual and joint inversion of MT and DC data, has been assessed initially using various sets of simulated synthetic keeping 10 population sizes/particles/agents and iteration of 1000 which has been executed 1000 providing 10^7^ inverted models, and finally demonstrated on field datasets extracted from different geological environments. We have also evaluated the bpdf and correlation matrix for uncertainty, non-uniqueness, and sensitivity of the posterior inverted results, which are shown through flow charts in two steps: STEP 1 for joint/individual inversion-based vPSOGWO algorithm and STEP 2 for posterior Bayesian pdf analysis has been applied to inverted models.

### Synthetic examples

In order to assess the performance of the proposed vPSOGWO method using Bayesian approach (pdf of those models are picked having more than 68.27% CI) over MT and DC datasets, we have procedure using 10% Gaussian noisy synthetic apparent resistivity data as follows: (i) individual inversion of the DC data, (ii) individual inversion of the MT data, and (iii) joint inversion of the DC and MT data. This simulated DC and MT synthetic data with 10% Gaussian noise was produced using forward modelling using Eqs. (1), (6), and (9). The inversion algorithm procedure uses a swarm size of 10, iteration of 1000, which has been executed 1000 times for calculating alternative models from the same datasets and comparing the inverted results with the available information, including error.

#### Example 1: Individual inversion of noisy synthetic DC resistivity-sounding data

**Figure 4:**
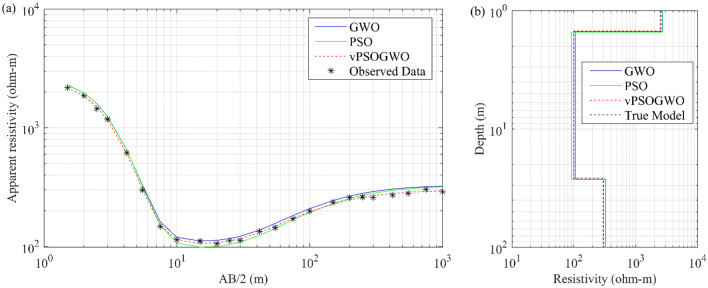
Three-layer noisy DC synthetic data: (**a**) observed apparent resistivity curve (*) and the best-fitted apparent resistivity curve using vPSOGWO, GWO, and PSO; (**b**) 1D mean model (bpdf > 68.27% CI) inverted by vPSOGWO (red), GWO (blue) and PSO (green) with a true model (black).

The Schlumberger apparent resistivity data distorted with 10% Gaussian noise is generated using a three-layered resistivity-depth model, as illustrated in Table 1. The experiment used the H-type curve with a high resistive layer of 2500 Ω-m, followed by 100 Ω-m and 300 Ω-m layers. Previous research conducted by Chandra et al.^15^ uses the same model with 2% Gaussian noise. The vPSOGWO algorithm has been presented here using simulated synthetic data. The inverted results are compared with the findings produced by Chandra et al.^15^ for PSO and GWO, as shown in Table 1. The comparison of synthetically generated data with computed apparent resistivity data using the vPSOGWO, GWO, and PSO techniques is shown in Fig. 4a. The 1D inverted resistivity-depth models are shown in Fig. 4b, with vPSOGWO algorithms having an RMS of 0.000476.

**Table 1:** Mean model with the level of uncertainty in the posterior utilizing individual inversion of DC synthetic data distorted with 10% noise from vPSOGWO, compared with results inverted by Chandra et al.^15^.

Layer parameters	True model	Search range		Inverted value			
		Lower	Upper	PSO (Chandra et al.^15^)	GWO (Chandra et al.^15^)	vPSOGWO (PDF = 100% CI)	vPSOGWO (PDF > 68.27% CI)
${\rho }_{1}$ (Ωm)	2500	1000	5000	2679.4 ± 32.67	2698.2 ± 24.17	2572.94 ± 16.34	2575.29 ± 2.64
${\rho }_{2}$ (Ωm)	100	1	1000	92.6 ± 40.5	104.8 ± 24.17	99.79 ± 1.72	100.05 ± 0.15
${\rho }_{3}$ (Ωm)	300	10	3000	324.53 ± 20.51	327.0 ± 8.94	297.44 ± 2.02	297.76 ± 0.24
${h}_{1}$ (m)	1.5	0.5	20	1.52 ± 0.04	1.5 ± 0.00	1.48 ± 0.01	1.48 ± 0.00
${h}_{2}$ (m)	25	1	100	25.1 ± 12.14	24.91 ± 0.97	24.30 ± 1.04	24.47 ± 0.10
Fitness				NRMS = 0.102	NRMS = 0.095	RMS = 0.0218

**Figure 5:**
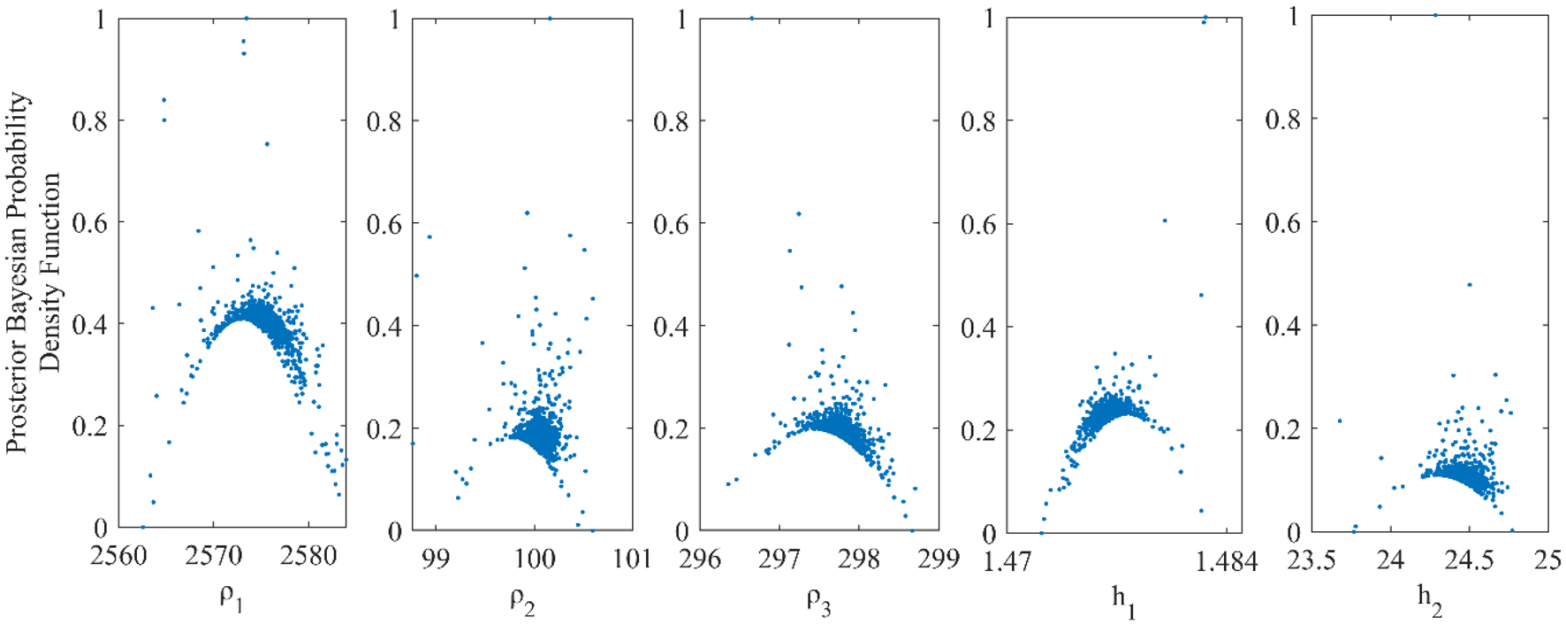
Bayesian posterior probability density function (*bpdf*) versus vPSOGWO inverted layer parameters for three-layered noisy DC synthetic data.

**Figure 6:**
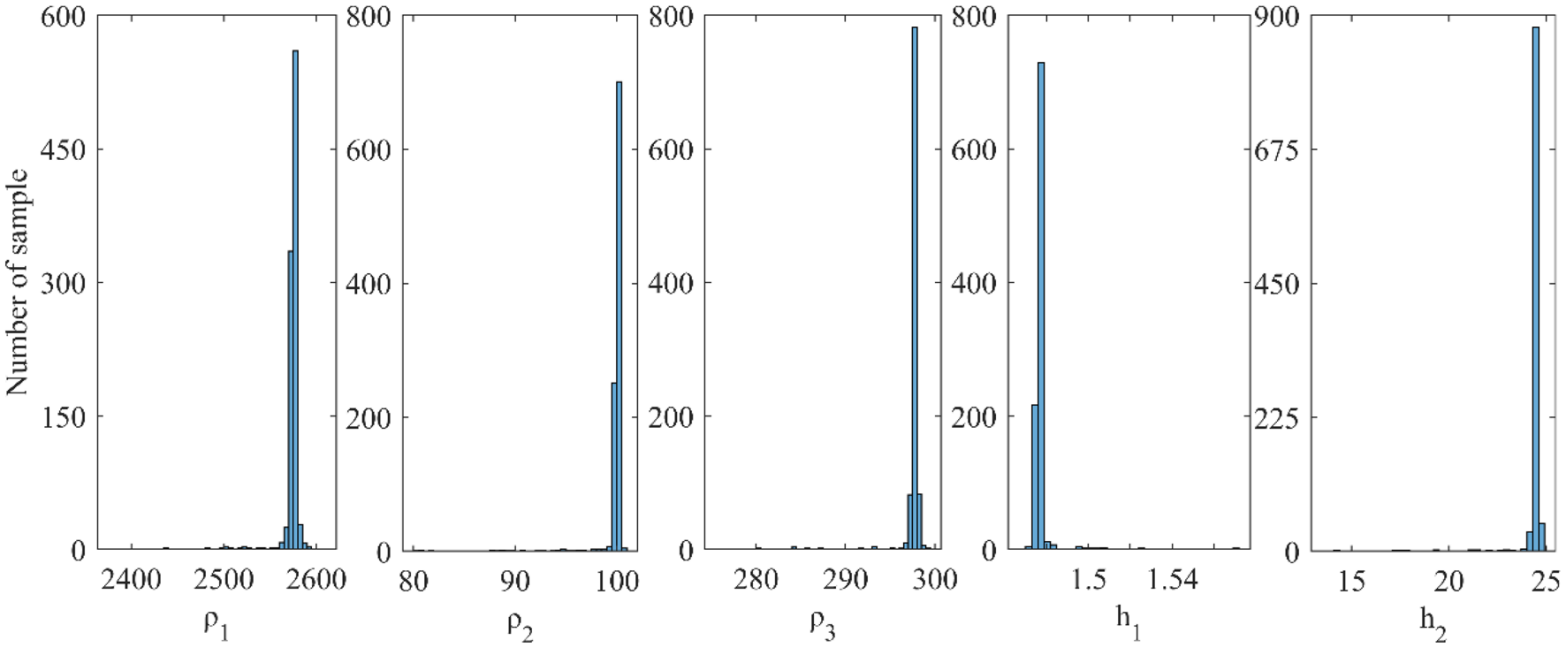
A number of samples versus vPSOGWO inverted layer parameters for three-layered noisy DC synthetic data.

We examined the inverted results from Fig. 4 and discovered that the outcomes obtained from vPSOGWO are consistently comparable with the already available results. However, the current vPSOGWO algorithm converges to a solution more precisely with high resolution and the least amount of uncertainty in model parameters, which is more accurate than the results simulated using GWO and PSO by Chandra et al.^15^ and well matched with a true model. Further, the accepted models (whose pdf is greater than 68.27% of CI) are created using the vPSOGWO technique are used to calculate the bpdf for understanding the resistivity model resolution and uncertainty (Fig. 5); the histogram for understanding the resistivity model sensitivity (Fig. 6). The apex of the curves (Fig. 4) shows how close the layer parameters occurs/most frequently to the actual model. Figure 5 shows that each layer parameter has been nicely resolved. The mean model, which demonstrates that most of the models lay quite similarly to/close to the correct model, determines the minimal uncertainty in the posterior.

As shown in Fig. 6, the highest resistivity values for the parameters ρ_1_, ρ_2_, and ρ_3_ are 97–99 Ωm (2 units), 285–289 Ωm (4 units) and 2570–2580 Ωm (10 units) out of the search ranges 1000–5000 Ωm (4000 units), 1–1000 Ωm (999 units), and 10–3000 Ωm (2990 units), respectively. While the greatest values for thicknesses parameters ℎ_1_ and ℎ_2_ are 1.43–1.44 m (0.01 unit) and 24.5–25 m (0.5 unit) out of the search ranges 0.5–20 m (19.5 unit) and 1–100 m (99 unit), respectively. As a result, compared to ρ_1_, ρ_2_, and ℎ_1_, the parameters ρ_3_ and ℎ_2_ exhibit greater fluctuation. The width of the histogram on the x-axis in Fig. 6 indicates that the DC data were more resolution/reliable to the shallow structure. Comparing the inverted model by Chandra et al.^15^, Table 1 shows that the thicknesses and resistivities parameters have little uncertainty. As a result, the vPSOGWO effectively lowers the uncertainty level and delivers the mean model more precisely.

Six different examples with different search ranges were explored in order to understand the stability of the hybrid vPSOGWO method over 10% noisy synthetic DC data. The results using vPSOGWO were compared to previous work by Chandra et al.^15^. Table 2 may be used to make the following conclusions: (i) when compared to the errors estimated by PSO and GWO, the error calculated by vPSOGWO is shown to have undergone negligible change, and (ii) the inverted models produced by vPSOGWO are almost similar in each case. This indicates that compared to PSO and GWO, the new vPSOGWO approach produces more accurate, stable, and accurate models with less error. We also noted that this strategy might produce outcomes more independent of the search spaces.

**Table 2:** Stability and sensitivity of 10% noisy synthetic DC resistivity sounding data.

Case	Layer parameters	True value	Search range	PSO^15^	GWO^15^	vPSOGWO
1	${\rho }_{1}$ (Ωm)	2500	1000–5000	2612.9	2747.5	2575.81
	${\rho }_{2}$ (Ωm)	100	10–1000	10	102.5	100.11
	${\rho }_{3}$ (Ωm)	300	100–3000	284.2	344.8	297.84
	${h}_{1}$ (m)	1.5	0–20	1.6	1.5	1.48
	${h}_{2}$ (m)	25	1–50	1.0	24.3	24.51
NRMS				0.5901	0.0819	0.0175
2	${\rho }_{1}$ (Ωm)	2500	1–5000	2691.4	2687.4	2576.25
	${\rho }_{2}$ (Ωm)	100	1–1000	106.4	104.7	100.07
	${\rho }_{3}$ (Ωm)	300	1–3000	325.6	320.8	297.83
	${h}_{1}$ (m)	1.5	0–20	1.5	1.5	1.48
	${h}_{2}$ (m)	25	1–50	26.1	24.3	24.48
NRMS				0.0619	0.0518	0.0179
3	${\rho }_{1}$ (Ωm)	2500	1500–5000	2696.5	2686.9	2576.16
	${\rho }_{2}$ (Ωm)	100	50–1000	112.9	103.7	100.07
	${\rho }_{3}$ (Ωm)	300	100–3000	339.8	321.6	297.80
	${h}_{1}$ (m)	1.5	1–20	1.5	1.5	1.48
	${h}_{2}$ (m)	25	10–50	33.9	23.9	24.48
NRMS				0.1828	0.0531	0.0178
4	${\rho }_{1}$ (Ωm)	2500	1500–3500	2692.7	2688.9	2575.65
	${\rho }_{2}$ (Ωm)	100	50–150	108.1	105.5	100.09
	${\rho }_{3}$ (Ωm)	300	200–400	328.1	325.5	297.78
	${h}_{1}$ (m)	1.5	1–2	1.5	1.5	1.48
	${h}_{2}$ (m)	25	15–35	27.9	25.9	24.49
NRMS				0.0833	0.0587	0.0177
5	${\rho }_{1}$ (Ωm)	2500	2000–5000	2693.9	2688.4	2575.65
	${\rho }_{2}$ (Ωm)	100	50–1500	109.2	104.8	100.10
	${\rho }_{3}$ (Ωm)	300	200–1000	338.1	323.6	297.81
	${h}_{1}$ (m)	1.5	1–50	1.5	1.5	1.48
	${h}_{2}$ (m)	25	15–100	32.3	24.8	24.50
NRMS				0.1522	0.0534	0.0176
6	${\rho }_{1}$ (Ωm)	2500	1–3000	2688.9	2690.2	2575.78
	${\rho }_{2}$ (Ωm)	100	1–200	109.0	107.4	100.09
	${\rho }_{3}$ (Ωm)	300	1–400	331.4	325.7	297.83
	${h}_{1}$ (m)	1.5	0–2	1.5	1.5	1.48
	${h}_{2}$ (m)	25	1–30	29.4	26.3	24.51
NRMS				0.1056	0.0653	0.0175

#### Example 2: Individual inversion of noisy synthetic MT resistivity-sounding data

The MT apparent resistivity data distorted with 10% Gaussian noise is generated using a typical continental crust containing a three-layered resistivity-depth model, as illustrated in Table 3. This continental geological model consists of a high resistive upper crust of 30,000 Ω-m) and 15 km thickness, followed by a moderately resistive middle crust of 5000 Ω-m resistivity and 18 km thickness, and a lower layer with low resistivity of 1000 Ω-m at a depth of 33 km.

**Table 3:** Mean model with the level of uncertainty in the posterior utilizing individual inversion of MT synthetic data distorted with 10% noise from vPSOGWO compared with results inverted by Chandra et al.^15^.

Layer parameters	True model	Search range		Inverted value			
		Lower	Upper	Chandra et al.^15^		Current inverted values	
				PSO	GWO	vPSOGWO (PDF = 100%CI)	vPSOGWO (PDF > 68.27% CI)
${\rho }_{1}$ (Ωm)	30,000	10,000	50,000	38,374.3 ± 10,857.3	31,215.8 ± 10,309.16	30,244.99 ± 18.86	30,245.02 ± 6.65
${\rho }_{2}$ (Ωm)	5000	100	25,000	4706.4 ± 432.98	4033.3 ± 576.23	5663.70 ± 51.96	5664.67 ± 20.80
${\rho }_{3}$ (Ωm)	1000	1	5000	996.1 ± 17.16	987.2 ± 3.45	986.75 ± 0.60	986.76 ± 0.21
${h}_{1}$ (m)	15,000	5000	25,000	14,690 ± 1340	16,170 ± 2980	14,230 ± 61	14,230 ± 18
${h}_{2}$ (m)	18,000	10,000	25,000	20,380 ± 1650	18,760 ± 1720	17,610 ± 43	17,610 ± 9.5
Fitness				NRMS = 0.050	NRMS = 0.049	RMS = 0.02543	

**Figure 7:**
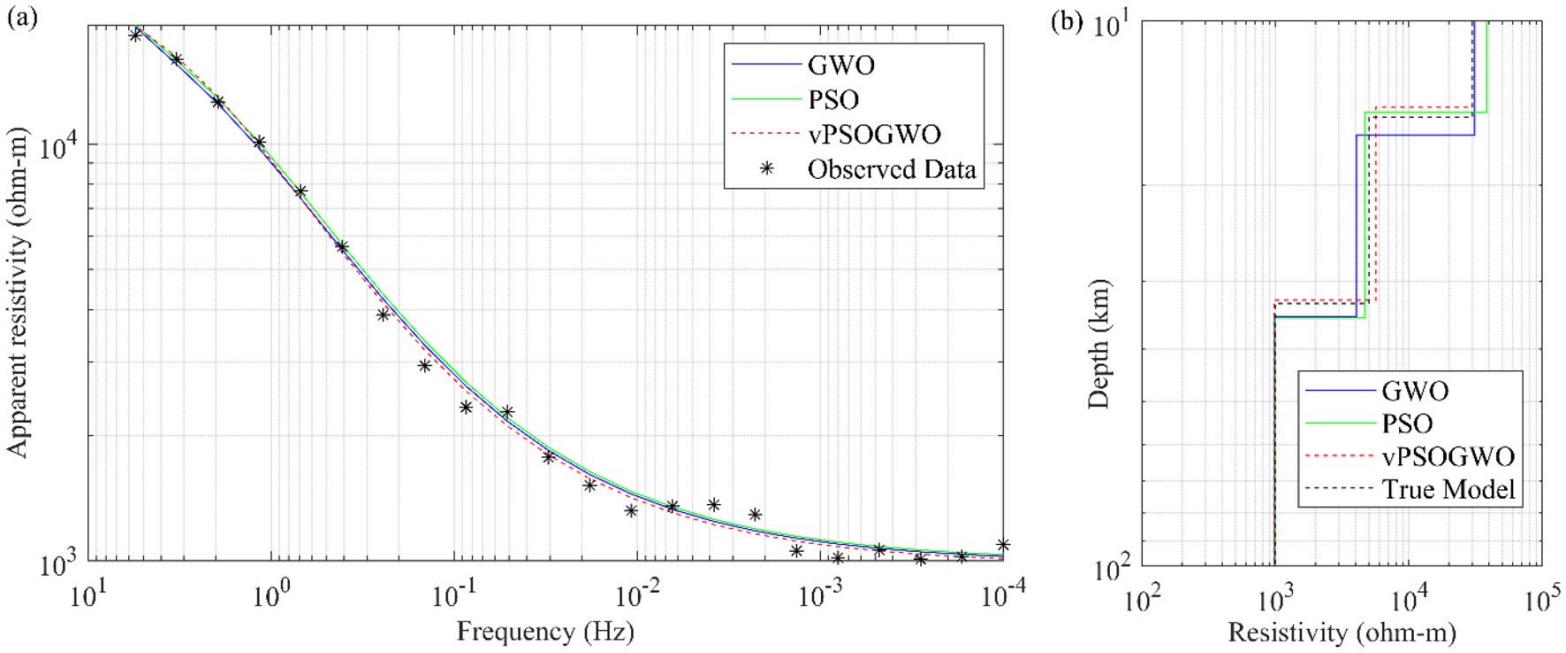
Three-layer noisy MT synthetic data: (**a**) observed apparent resistivity curve (*) and the best-fitted apparent resistivity curve using vPSOGWO, GWO, and PSO; (**b**) 1D mean model (bpdf > 68.27% CI) inverted by vPSOGWO (red), GWO (blue) and PSO (green) with a true model (black).

Previous research by Chandra et al.^15^ using the same model was conducted with 2% Gaussian noise and its outcome, along with search ranges and the inverted results, as shown in Table 3. The vPSOGWO has been presented here using simulated MT synthetic data, and the inverted results are compared with the findings produced by Chandra et al.^15^, as shown in Table 3. The comparison of synthetically generated data with computed apparent resistivity data is shown in Fig. 7a. The 1D inverted resistivity-depth model is shown in Fig. 7b, with the actual model incorporating the RMS error of 0.02543.

We examined the inverted results, as given in Fig. 7, and discovered that the outcomes obtained from vPSOGWO are comparable with the available results. However, the current vPSOGWO algorithm converges to a solution more precisely with high resolution and the least amount of uncertainty, which is more accurate than the results inverted by GWO, PSO published results and well correlated with a true model.

**Figure 8:**
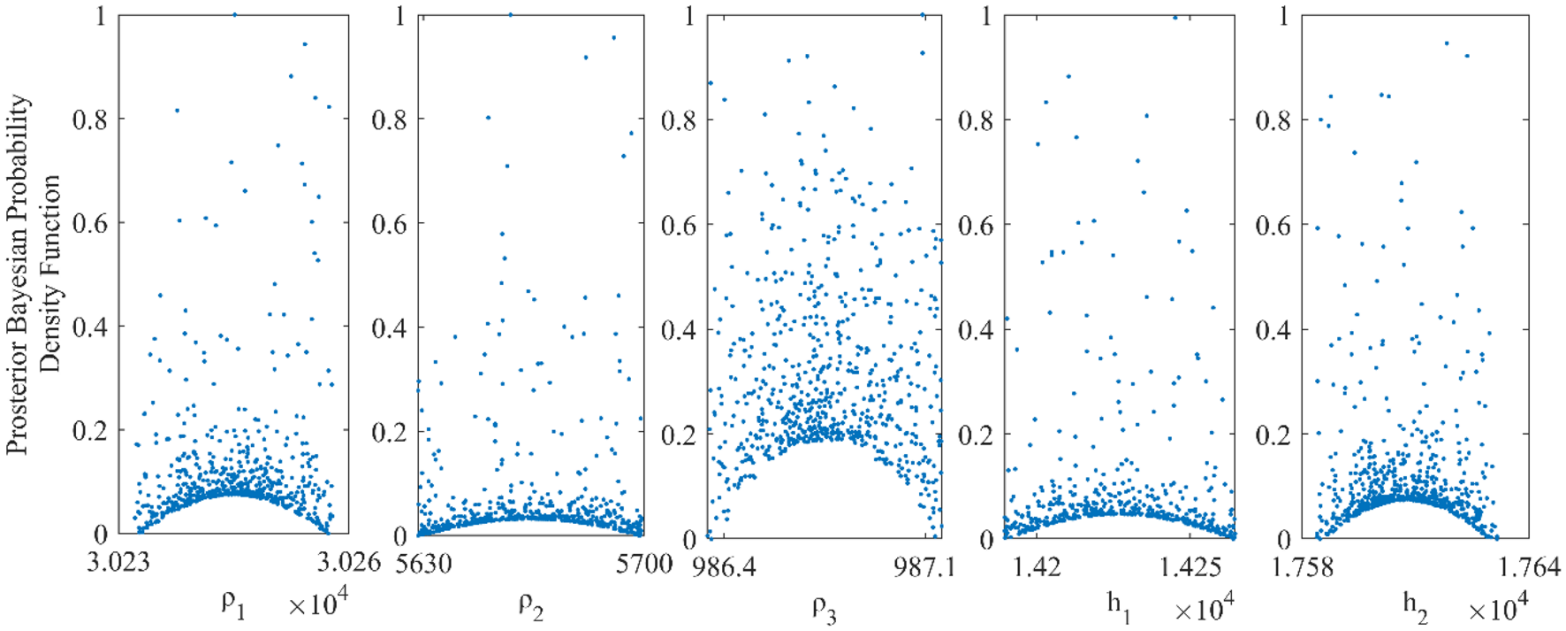
Bayesian posterior probability density function (*bpdf*) versus vPSOGWO inverted layer parameters for three-layered noisy MT synthetic data.

**Figure 9:**
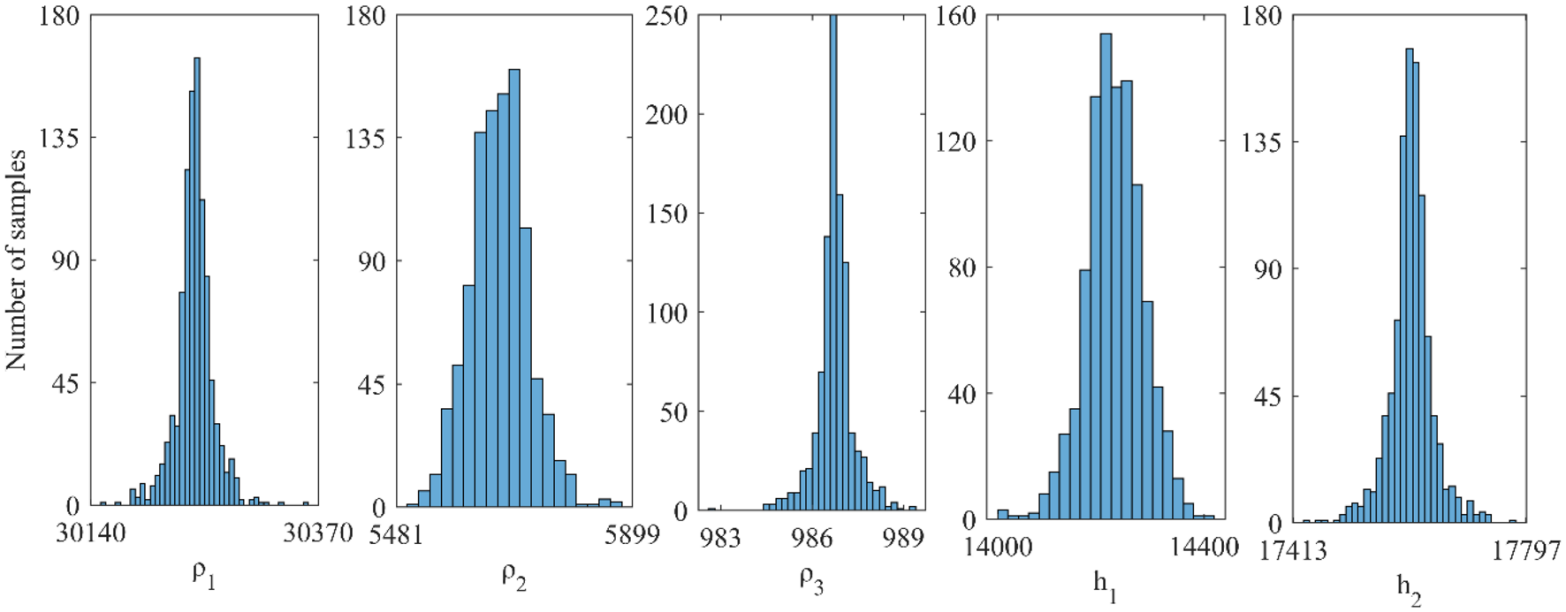
A number of samples versus vPSOGWO inverted layer parameters for three-layered noisy MT synthetic data.

Further, the response of the inverted result by vPSOGWO, the accepted models (whose pdf is greater than 68.27% of CI) are created, and calculate the bpdf values for understanding the resistivity model resolution and uncertainty (Fig. 8) and the histogram for understanding the resistivity model sensitivity (Fig. 7). The apex of the curves (Fig. 8) shows how close the layer parameters occurs/most frequently to the actual model. Figure 9 shows that the resistivity of the first and third layers is strongly resolved for each dataset; however, the intermediate layer model parameter has low resolution. Because the equivalence problem has evidence supporting it. The mean model, which demonstrates that the majority of the models lay quite similarly to/close to the correct model, determines the uncertainty in the posterior.

The maximum resistivity values for the parameters ${\rho }_{1}$, ${\rho }_{2}$, and ${\rho }_{3}$ are 30,240–30,260 Ωm (20 units), 5621–5699 Ωm (78 units), and 987–988 Ωm (1 unit) out of the search ranges 10,000–50,000 Ωm (40,000 units), 100–25,000 Ωm (24,900 units), and 1–5000 Ωm (4999 units), respectively. While the largest values for thicknesses parameters ℎ_1_ and ℎ_2_ are, 14,170–14,238 m (68 units) and 17,583–17,613 m (51 units) out of the search ranges 0.5–20 m (19.5 units) and 1–100 m (99 units), respectively.

As a result, compared to ρ_1_, ρ_2_, and ℎ_1_, the parameters ρ_3_ and ℎ_1_ exhibit more significant fluctuation. The MT data were more trustworthy and had a higher resolution for the deeper structure, as seen by the breadth of the histogram on the x-axis in Fig. 9. Table 3 demonstrates that there is less uncertainty in the thickness and resistivity parameters compared to the inverted model by Chandra et al.^15^. The vPSOGWO successfully reduces the level of uncertainty as a result, and gives the mean model with greater precision.

We also performed the exercise using MT data over 10% noisy synthetic MT data, which is similar to the DC resistivity data presented in Table 2, in order to examine and understand the stability of the vPSOGWO method. Examining the vPSOGWO inverted models, the findings were compared with findings analysed by Chandra et al.^15^. Table 4 shows that while the inverted model created by vPSOGWO in each case was almost identical, there was no substantial variance in the estimated error, but the error, variation in error, and variation in model identified by PSO and GWO are comparably larger. From Tables 2 and 4, it can be inferred that the current vPSOGWO technique is capable of balancing the exploitation capability of PSO and exploration capability of GWO23. As a result, the current algorithm provides a more accurate and stable solution with the least amount of error that is closer to the actual model than the PSO and GWO.

**Table 4:** Stability and sensitivity of 10% noisy synthetic MT data.

Case	Layer parameters	True value	Search range	PSO^15^	GWO^15^	vPSOGWO
1	${\rho }_{1}$ (Ωm)	30,000	5000–50,000	49,957.1	25,517.3	29,931.73
	${\rho }_{2}$ (Ωm)	5000	1000–25,000	4754.7	4039.4	5641.31
	${\rho }_{3}$ (Ωm)	1000	50–5000	987.2	986.3	975.40
	${h}_{1}$ (m)	15,000	5000–25,000	13,383.5	16,897.1	14,392
	${h}_{2}$ (m)	18,000	10,000–25,000	21,432.2	18,436.2	18,123
NRMS				0.3140	0.1233	0.0612
2	${\rho }_{1}$ (Ωm)	30,000	100–50,000	25,958.2	31,545.5	29,932.41
	${\rho }_{2}$ (Ωm)	5000	1000–20,000	4429.5	4333.2	5631.63
	${\rho }_{3}$ (Ωm)	1000	1–5000	1028.3	989.9	975.32
	${h}_{1}$ (m)	15,000	5000–25,000	16,200.0	15,374.4	14,408
	${h}_{2}$ (m)	18,000	10,000–25,000	17,500.5	19,757.4	18,134
NRMS				0.0885	0.0784	0.0603
3	${\rho }_{1}$ (Ωm)	30,000	10–50,000	49,890.8	19,159.8	29,929.64
	${\rho }_{2}$ (Ωm)	5000	10–20,000	4703.4	2904.5	5635.90
	${\rho }_{3}$ (Ωm)	1000	10–5000	982.4	980.8	975.37
	${h}_{1}$ (m)	15,000	10,000–25,000	13,473.0	21,197.5	14,402
	${h}_{2}$ (m)	18,000	10,000–25,000	21,425.6	15,646.3	18,111
NRMS				0.3131	0.3144	0.0607
4	${\rho }_{1}$ (Ωm)	30,000	100–50,000	31,545.5	48,149.9	29,932.67
	${\rho }_{2}$ (Ωm)	5000	1000–20,000	5448.3	4333.2	5629.68
	${\rho }_{3}$ (Ωm)	1000	1–5000	1002.2	989.9	975.36
	${h}_{1}$ (m)	15,000	5000–25,000	15,374.4	12,265.2	14,403
	${h}_{2}$ (m)	18,000	10,000–25,000	21,522.4	19,757.4	18,122
NRMS				0.0996	0.2921	0.0602
5	${\rho }_{1}$ (Ωm)	30,000	25,000–50,000	44,017.0	37,231.1	29,926.91
	${\rho }_{2}$ (Ωm)	5000	1000–20,000	4749.5	4480.9	5635.87
	${\rho }_{3}$ (Ωm)	1000	500–5000	988.8	989.0	975.37
	${h}_{1}$ (m)	15,000	10,000–25,000	13,652.9	14,549.2	14,400
	${h}_{2}$ (m)	18,000	15,000–30,000	21,104.7	20,491.1	18,123
NRMS				0.2275	0.1335	0.0607
6	${\rho }_{1}$ (Ωm)	30,000	25,000–35,000	28,877.0	25,691.5	29,932.70
	${\rho }_{2}$ (Ωm)	5000	1000–10,000	4153.2	4108.9	5640.98
	${\rho }_{3}$ (Ωm)	1000	500–5000	987.2	987.5	975.40
	${h}_{1}$ (m)	15,000	10,000–20,000	16,068.1	16,734.4	14,393
	${h}_{2}$ (m)	18,000	15,000–25,000	19,277.3	18,465.9	18,121
NRMS				0.0898	0.1154	0.0612

#### Example 3: Joint inversion of noisy DC and MT resistivity-sounding data

**Figure 10:**
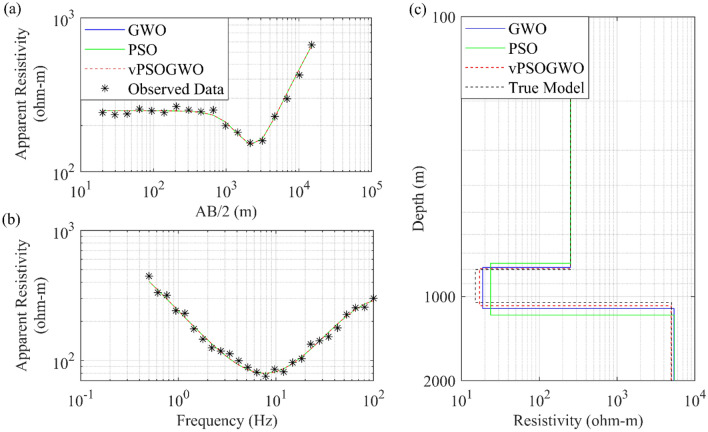
Three-layer noisy DC and MT synthetic apparent resistivity data: (**a**) observed (*) and the best-fitted curves using vPSOGWO, GWO, and PSO; (**b**) 1D mean model (bpdf > 68.27% CI) inverted by vPSOGWO (red), GWO (blue) and PSO (green) with an actual model (black).

To investigate the efficacy of the multi-parametric joint inversion approach, synthetic responses over a sedimentary basin covered by a basaltic trap were constructed for DC and MT datasets. To approximate the field condition, we contaminated these datasets with 10% Gaussian noise, as illustrated in Fig. 10, which shows the results of numerical tests on a sample geological model. Here, a 250 m thick layer of low resistive sediments is studied, sandwiched between a 250 Ω-m resistive overburden layer of 800 m thick basalts and a high resistive granitic basement. Followed by joint inversion of MT and DC datasets using vPSOGWO, GWO, and PSO algorithms were performed and analyzed without any starting estimations over result of one thousand runs. Finally, the joint inversion results are examine together to determine the overall improvement in parameter estimations. Thus found to have an excellent estimates of all layer parameters using vPSOGWO, GWO, and PSO (Fig. 10) with error 0.0899, 0.1658 and 0.3816, and their associated time 1.94 Sec 1.84 Sec and 1.85 Sec per model, respectively (Table 5). The mean model obtained from vPSOGWO are reasonably near to their true model and better than results analyzed by Manglik et al.^39^. These findings show that the equivalency problem related to the sedimentary layer has been much minimized. The current work was executed in MATLAB R2020a with CPU cluster having Processor: 2 × Intel Xeon Gold 6242 @2.8Ghz, 192 GB DDR4-2933 RAM, 200 TB PFS storage.

**Table 5:** Mean model with the level of uncertainty in the posterior utilizing individual inversion of MT synthetic data distorted with 10% noise from vPSOGWO compared with results inverted by Manglik et al.^39^.

Layer parameters	True model	Search range		Inverted value					
		Lower	Upper	Manglik et al.^39^			Current inverted values (PDF > 68.27% CI)		
				Model -1	Model -2	Model -3	PSO	GWO	vPSOGWO
${\rho }_{1}$ (Ωm)	250	100	500	255*.*4 ± 27*.*4	254*.*8 ± 27*.*3	255 ± 27*.*3	252.01 ± 0.35	250.88 ± 0.19	250.58 ± 0.30
${\rho }_{2}$ (Ωm)	15	1	50	18*.*9 ± 0*.*84	17*.*2 ± 0*.*76	17*.*7 ± 0*.*79	23.67 ± 1.39	18.71 ± 0.88	17.00 ± 0.65
${\rho }_{3}$ (Ωm)	5000	1000	10,000	5675 ± 1650	5670 ± 1647	5672 ± 1648	5450.38 ± 136.48	5397.53 ± 277.68	4955.32 ± 132.93
${h}_{1}$ (m)	800	500	1500	772*.*4 ± 88*.*1	781*.*8 ± 88*.*1	778*.*8 ± 88*.*1	759.53 ± 6.96	785.60 ± 4.52	759.53 ± 3.57
${h}_{2}$ (m)	250	100	500	317*.*5 ± 12*.*7	286*.*2 ± 11*.*5	296*.*1 ± 11*.*9	404.81 ± 25.46	315.98 ± 15.45	285.33 ± 11.13
Fitness (NRMS)							0.3816	0.1658	0.0899
CPU time (s)							1.85	1.84	1.94

**Figure 11:**
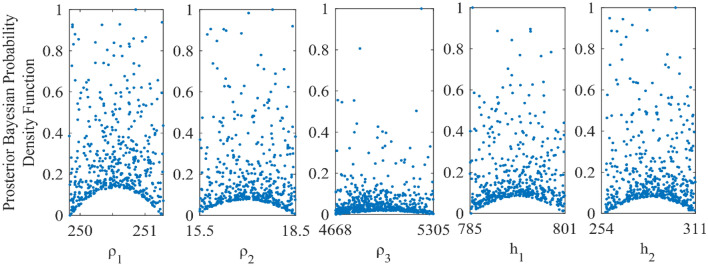
Bayesian posterior probability density function (*bpdf*) versus vPSOGWO inverted layer parameters for three-layered noisy DC and MT synthetic datasets.

**Figure 12:**
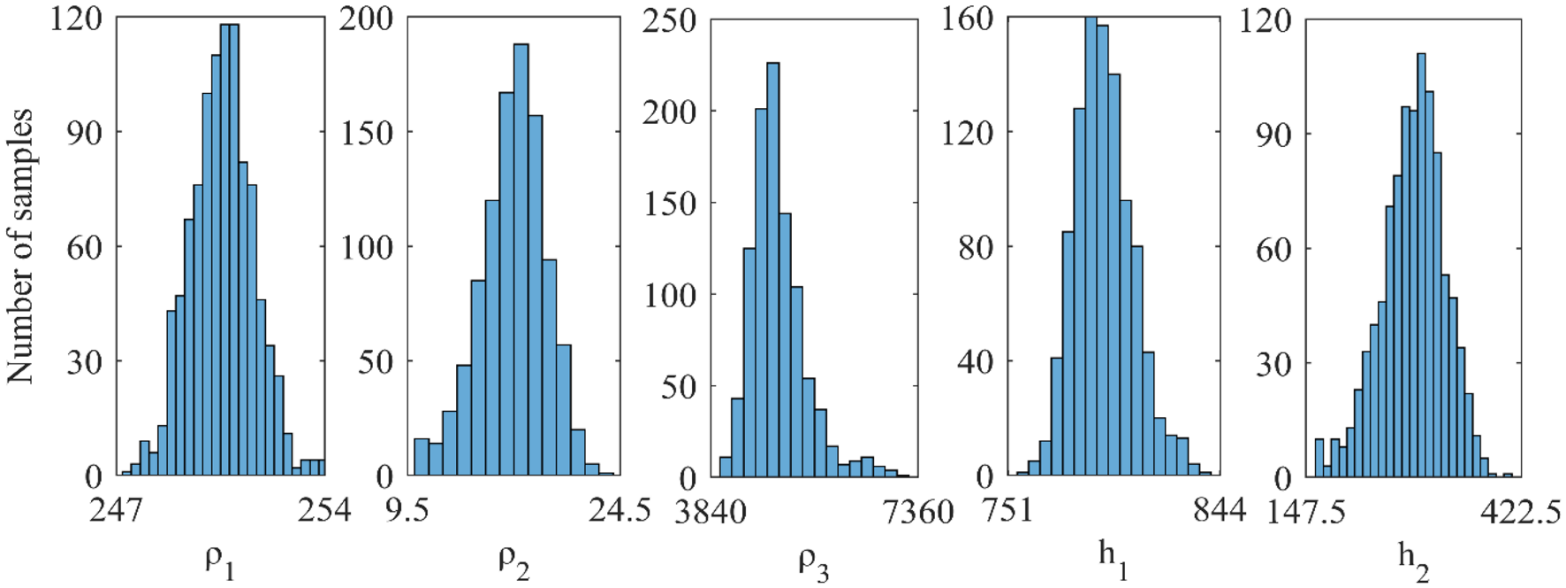
A number of samples versus vPSOGWO inverted layer parameters for three-layered noisy DC and MT synthetic datasets.

In addition, the response of the inverted result by vPSOGWO, accepted models (whose pdf is greater than 68.27% of CI) are created. We have also calculated the bpdf values for understanding the resolution and uncertainty in the models (Fig. 11) and prepared histogram for understanding the sensitivity in the layer model (Fig. 12). The peak of the curves (Fig. 12) depicts how closely the layer parameters occur to the real model. The posterior's uncertainty is determined by the mean model, which shows that most models fit pretty close to the real model.

In Fig. 12, the number of samples is shown against the layer parameters that were inverted by vPSOGWO and the x-axis shows the width of the histogram. This show a remarkable variation in the parameters ρ_3_ and ℎ_1_, as opposed to ρ_1_, ρ_2_, and ℎ_1_. The DC and MT data were more reliable for better resolution in shallow and deeper structure, respectively. In comparison to the inverted model by Manglik et al.^39^, Table 5 shows less amount of uncertainty in the thickness and resistivity parameters. As a consequence, the vPSOGWO successfully lowers the amount of uncertainty and produces a mean model with higher precision.

### Field examples

We have ultimately deployed the newly created strategy vPSOGWO to six sets of field data from various geological sequences while preserving the same swarm size and model number, based on the efficacy of the approach. These datasets include: These datasets are: (i) DC resistivity sounding data from Digha, West Bengal, India; (ii) DC resistivity sounding data from New Brunswick, Canada; (iii) MT resistivity sounding data from Sundar Pahari, Jharkhand, India; (iv) MT sounding data from the Puga valley, Ladakh, India; (v) DC and MT data over Broken Hill, Australia; and (vi) DC and MT data over Central Puga valley, Ladakh, India. Each case's outcomes were compared to the published inverted models and borehole information.

#### Example 1: Individual inversion of DC resistivity sounding data from Digha, India

The first field sample uses Schlumberger resistivity sounding data from Digha, Medinipur (WB), which was digitized from Patra and Bhattacharya^40^, where alternating sedimentary rock of sand and clay is present with a thin alluvium layer above it. The purpose of gathering this information was to look into the influx of salinity and identify any pockets of it. Sen et al.^41^ demonstrate the simulating annealing technique (SA) using the same field example and explain the presence of an alluvial layer with a resistivity of 32.2 ± 0.93 Ω-m and a thickness of 3.99 ± 0.13 m. This layer indicates a highly conductive zone with a resistivity of 2.68 ± 0.43 Ω-m and a thickness of 19.8 ± 1.13 m. In this case, PSO, GWO, and vPSOGWO inversions were carried out using the same search parameters as Sen et al.^41^.

**Figure 13:**
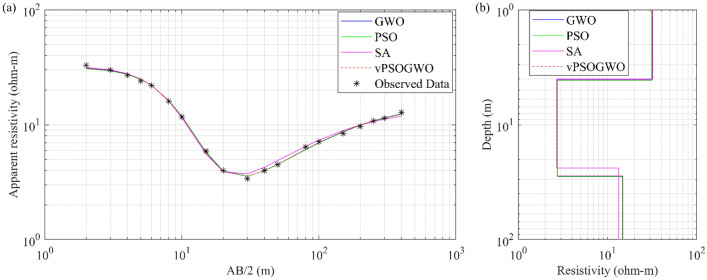
Three-layers DC field data over Digha, Medinipur (WB), India: (**a**) observed (*) and the best-fitted apparent resistivity curves using GWO, PSO, SA, and vPSOGWO; (**b**) 1-D mean model (bpdf > 68.27% CI) inverted by vPSOGWO (red), SA (magenta), PSO (Green) and GWO (Blue).

Figure 13a illustrates the measured and estimated apparent resistivity data, while Fig. 13b displays the inverted 1D depth model produced by the PSO, GWO, and vPSOGWO algorithms with mean square errors of 1.6515e−4, 1.5174e−4, and 1.5173e−4, respectively (Table 6). It is evident from Table 5 and Fig. 13 that the standard deviation of the model parameters for vPSOGWO is relatively low compared to other algorithms. As a result, the current vPSOGWO technique is compatible with providing a better and more accurate solution with the least degree of model uncertainty than PSO, GSA, and published results by Sen et al.^41^, which is closer and in good agreement with the known lithological log which is provided in Patra and Bhattacharya^40^.

**Table 6:** Mean model with the level of uncertainty in the posterior utilizing individual inversion of DC data from PSO, GWO, vPSOGWO, compared with results inverted by Sen et al.^41^ over Digha, Medinipur (WB), India.

Layer parameters	Search range		Sen et al.^41^	Inverted values (PDF > 68.27% CI)		
	Lower	Upper	SA	PSO	GWO	vPSOGWO
${\rho }_{1}$ (Ωm)	10	100	32.2 ± 0.93	31.32 ± 1.15	31.72 ± 0.20	31.73 ± 0.04
${\rho }_{2}$ (Ωm)	0.1	10	2.68 ± 0.43	2.77 ± 0.42	2.74 ± 0.21	2.74 ± 0.03
${\rho }_{3}$(Ωm)	5	20	13.4 ± 1.13	14.69 ± 2.08	14.82 ± 0.15	14.81 ± 0.03
${h}_{1}$ (m)	1	15	3.99 ± 0.13	4.11 ± 0.19	4.06 ± 0.07	4.07 ± 0.013
${h}_{2}$ (m)	1	50	19.8 ± 1.13	24.21 ± 6.17	23.85 ± 1.91	23.80 ± 0.37
Fitness (RMS)			Not available	1.6515e−4	1.5174e−4	1.5173e−4

#### Example 2: Individual inversion of DC resistivity sounding data from New Brunswick, Canada

**Figure 14:**
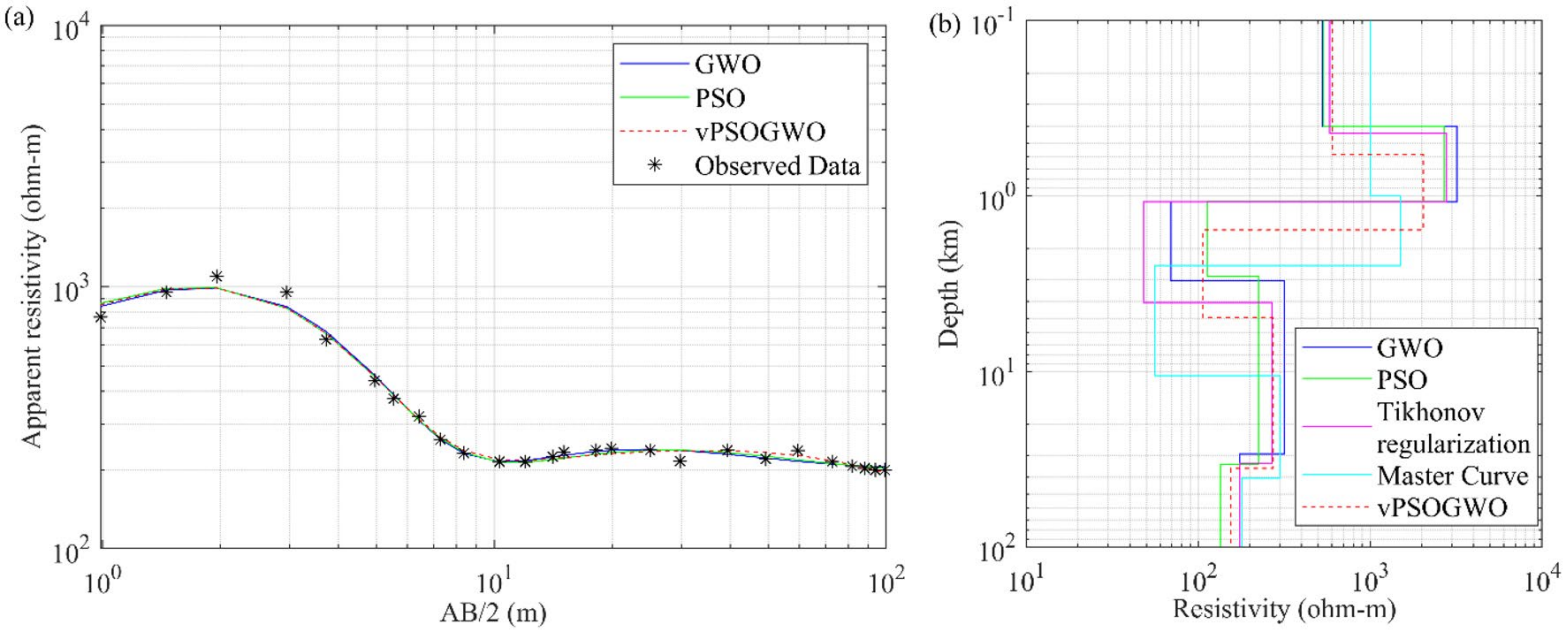
Five-layers DC field data over Chatham, New Brunswick, Canada: (**a**) observed (*) and the best-fitted apparent resistivity curve using GWO, PSO, and vPSOGWO; (**b**) 1-D mean model (bpdf > 68.27% CI) inverted by vPSOGWO (red), Master Curve (cyan), Tikhonov regularization (magenta), PSO (green) and GWO (blue).

Another field example over a five-layers model utilizing Schlumberger resistivity sounding data was obtained for conducting the hydrological research in Chatham, New Brunswick, Canada^41^. The resistivity parameters for various layer formations with hydrological features/characteristics are shown in Table 7. Table 8 displays the search range and inverted results for the master curve^42^, Tikhonov regularization^43^, PSO^42^, GWO^42^, and presented vPSOGWO techniques. Figure 14a depicts the observed and computed apparent resistivity data, while Fig. 14b shows the 1D depth model that has been inverted using all of the aforementioned strategies. Table 7 demonstrates that the hybrid approach well agrees with the lithology and current geological information. Table 8 demonstrates that the hybrid method has a lower error rate than other algorithms.

**Table 7:** Resistivity characteristic of different rock types (after Roy and Elliot^42^).

Geological formation	Hydrogeological characteristics	Resistivity (Ω-m)
Overburden (sandy soil and boulders)	Dry	2000–12,000
Shale, siltstone, and clay	Aquiclude	25–60
Sandy shales	Aquitard	80–120
Shaly sandstones	Poor aquifer	200–240
Sandstone (saturated)	Good aquifer	280–400
Sandstone (unsaturated)	Dry	750–1200
Sandstone (cemented)	Aquifer; probably of low yield	600–900
Saltwater contaminated formations	No use or restricted use	Less than 120

**Table 8:** Mean model with the level of uncertainty in the posterior utilizing individual inversion of MT data from vPSOGWO, compared with results inverted by Master curve^42^, Tikhonov regularization^42^ and Chandra et al.^15^ over Chatham, New Brunswick, Canada.

Layer parameters	Search range		Inverted value				
	Lower	Upper	Master curve (Roy and Elliot^42^)	Tikhonov regularization (Roy^43^)	Chandra et al.^15^		vPSOGWO (PDF > 68.27% CI)
					PSO	GWO	
${\rho }_{1}$ (Ωm)	100	5000	1000	581	537.13	528.97	602.87 ± 106.08
${\rho }_{2}$ (Ωm)	100	5000	1500.00	2794	2705.08	3199.85	2042.39 ± 512.42
${\rho }_{3}$ (Ωm)	1	200	56.00	48	112.72	69.49	106.93 ± 14.69
${\rho }_{4}$ (Ωm)	50	500	300.00	270	223.46	318.09	271.28 ± 27.13
${\rho }_{5}$ (Ωm)	100	500	180.00	175	134.34	174.93	154.56 ± 17.67
${h}_{1}$ (m)	0.01	2	1.00	0.44	0.40	0.40	0.58 ± 0.30
${h}_{2}$ (m)	0.1	5	1.50	0.64	0.67	0.68	0.98 ± 0.33
${h}_{3}$ (m)	1	15	8.05	2.98	1.81	1.98	3.37 ± 0.84
${h}_{4}$ (m)	10	100	30.0	29.22	30.86	26.18	30.63 ± 8.29
Fitness (NRMS)					NRMS = 0.073	NRMS = 0.058	RMS = 0.0224

#### Example 3: Individual inversion of MT resistivity sounding data from Sundar Pahari, Dhanbad, India

**Figure 15:**
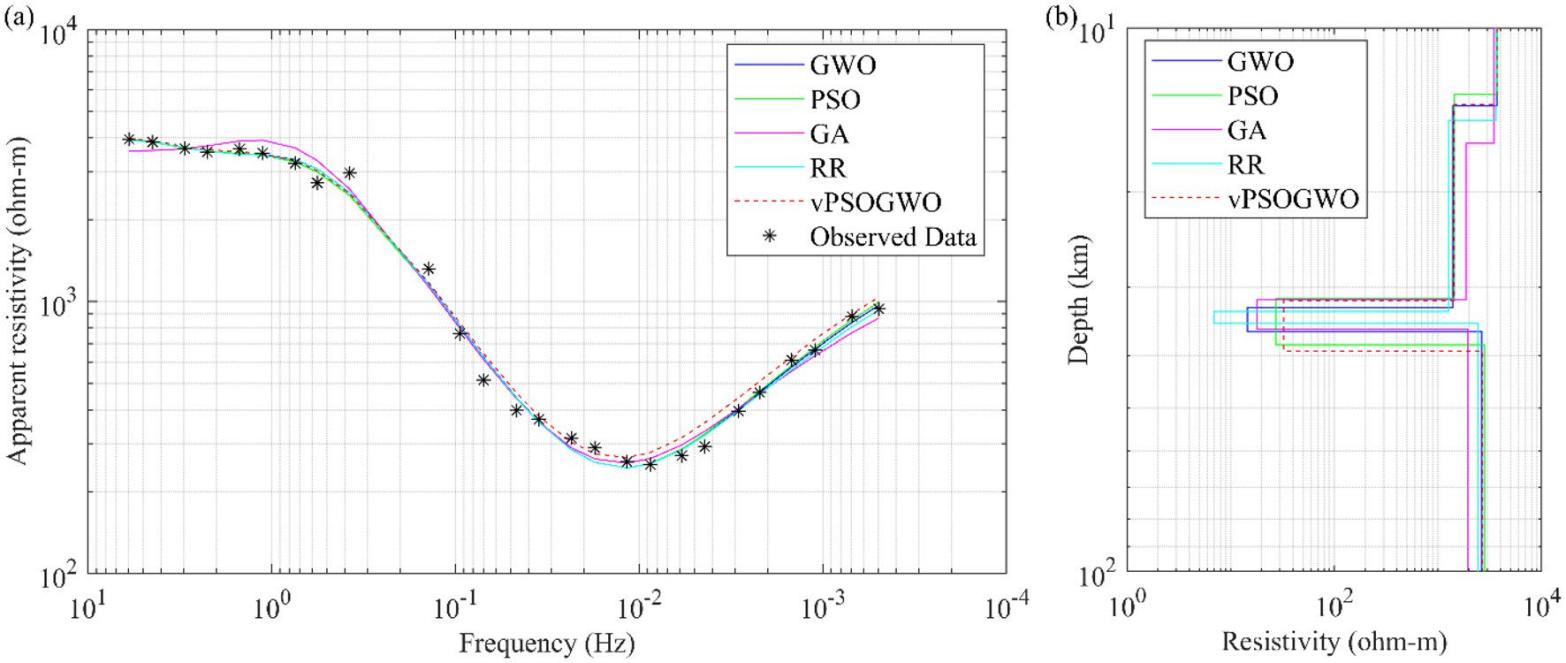
Four-layers MT field data over Sundar Pahari, Dhanbad, India: (**a**) observed (*) and the best-fitted apparent resistivity curve using GWO, PSO, GA, RR, and vPSOGWO; (**b**) 1-D mean model (bpdf > 68.27% CI) inverted by vPSOGWO (red), RR (cyan), GA (magenta), PSO (Green) and GWO (Blue).

MT field apparent resistivity data from the Chhotanagpur gneissic complex in Sundar Pahari, Dhanbad^44^ were used as the first example, and their usefulness was evaluated using the vPSOGWO algorithm. A conductive zone is present because of a noticeable fall in resistivity at about 0.01 Hz, which is explained by the geology of the research area, which has a resistant layer equivalent to granite gneiss at high frequencies. To construct the 1D resistivity depth model of the earth’s subsurface over the study area, the acquired apparent resistivity was inverted aforementioned algorithm, and the inverted results were compared with results obtained from the Ridge Regression (RR)^44^, Genetic Algorithm (GA)^44^ and PSO^44^ as shown in Table 9. Figure 15a shows the observed and the calculated apparent resistivity curves, whereas Fig. 15b depicts the 1D depth model for inverted models derived from different algorithms.

**Table 9:** Mean model with the level of uncertainty in the posterior utilizing individual inversion of MT data from the findings of the vPSOGWO, compared with results inverted from Shaw and Shalivahan^44^ and Chandra et al.^15^ over Sundar Pahari, Dhanbad, India.

Layer parameters	Search range		Inverted values				
	Lower	Upper	Shaw and Shalivahan^44^			GWO (Chandra et al.^15^)	vPSOGWO (PDF > 68.27% CI)
			RR	GA	PSO		
${\rho }_{1}$ (Ωm)	1000	10,000	3654.0	3480	3749.4	3713.58	3553.46 ± 598.93
${\rho }_{2}$ (Ωm)	100	2500	1272.1	1870.0	1447.0	1392.34	996.30 ± 391.78
${\rho }_{3}$ (Ωm)	10	300	6.9	18.0	27.3	14.53	47.21 ± 23.22
${\rho }_{4}$ (Ωm)	1000	5000	2417.3	1960.0	2806.1	2649.40	2705.99 ± 39.65
${h}_{1}$ (km)	1	50	14.76	16.30	13.23	13.91	19.58 ± 4.54
${h}_{2}$ (km)	1	50	18.50	15.30	18.21	18.75	20.32 ± 3.63
${h}_{3}$ (km)	1	15	1.67	4.20	6.80	3.58	8.31 ± 1.58
Fitness			NRMS = 0.012	NRMS = 0.014	NRMS = 0.012	NRMS = 0.101	RMS = 0.035

#### Example 4: Individual inversion of MT sounding data from Western Puga valley, Ladakh, India

**Figure 16:**
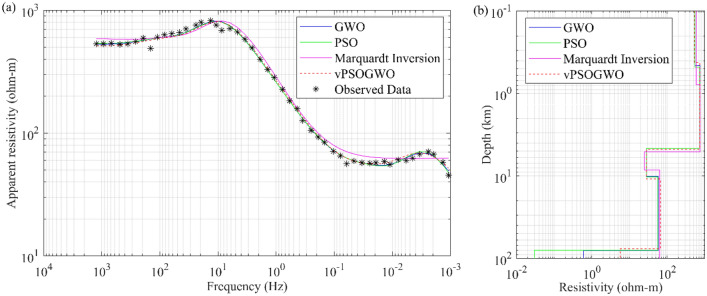
Five-layers MT field data over Western Puga valley geothermal field, Ladakh, India: (**a**) observed (*) and the best-fitted apparent resistivity curves using GWO, PSO, Marquardt^44^, and vPSOGWO; (**b**) 1-D mean model (bpdf > 68.27% CI) inverted by vPSOGWO (red), Marquardt (magenta), PSO (green) and GWO (blue).

Another set of field MT apparent resistivity data with a frequency range of 0.001 to 1200 Hz has been collected to test the viability of the vPSOGWO, GWO, and PSO techniques in some different and challenging geothermal and geological setups, such as the south of the Karakorum Fault in the Puga valley, Ladakh^45^ (Station B05). Puga valley is a notable geothermal province in the northwest Himalayan belt area, located in the southeast of the Ladakh Union territory of India at the height of 4400 m above mean sea level near the meeting point of the Indian and Asian plates. The hot springs in Puga Valley have an average temperature of 84 °C and are renowned for containing deposits of borax and sulphur^45^. Many geoscientists carried out exploration studies to find out whether a geothermal anomaly, shallow and deeper reservoir features and geothermal characteristics existed^46–49^. Using the aforementioned techniques, the apparent resistivity data was inverted here, and the inverted findings were seen together with the search range taken in this investigation, as given in Table 10. Figure 16a displays the measured and computed apparent resistivity curves, whereas Fig. 16b shows the 1D depth model derived by the vPSOGWO, GWO, PSO, and Marquardt algorithms by Harinarayana et al.^45^. After analyzing Table 10 and Fig. 16b, we discovered the two findings below: (i) The current techniques provide an additional layer of 65.68 km thickness that has a geological significance and was not resolved by Harinarayana et al.^45^; (ii) the crustal thickness over the study area is 76.58, 80.18, and 79.86 km inverted by vPSOGWO, GWO, and PSO algorithms, respectively.

**Table 10:** Mean model with the level of uncertainty in the posterior utilizing individual inversion of MT data from the findings of the PSO, GWO, vPSOGWO, compared with results inverted by Harinarayana et al.^45^ over Western Puga valley geothermal field, Ladakh, India.

Layer parameters	Search range		Marquardt inversion^44^	Inverted value (PDF > 68.27% CI)		
	Lower	Upper		PSO	GWO	vPSOGWO
${\rho }_{1}$ (Ωm)	100	1000	588.60	547.66 ± 44.00	540.64 ± 40.42	523.11 ± 21.90
${\rho }_{2}$ (Ωm)	100	1000	743.03	734.19 ± 62.65	741.30 ± 24.72	731.27 ± 12.48
${\rho }_{3}$ (Ωm)	1	50	24.48	28.28 ± 4.74	27.85 ± 1.47	28.05 ± 0.76
${\rho }_{4}$ (Ωm)	10	1000	62.93	56.86 ± 269.00	58.16 ± 36.73	66.68 ± 26.22
${\rho }_{5}$ (Ωm)	0.01	10	NA	0.03 ± 3.08	0.60 ± 2.75	5.69 ± 1.33
${h}_{1}$ (km)	0.1	1	0.78	0.48 ± 0.21	0.46 ± 0.15	0.42 ± 0.73
${h}_{2}$ (km)	3	6	4.35	4.14 ± 0.45	4.17 ± 0.19	4.32 ± 0.11
${h}_{3}$ (km)	1	10	3.31	5.66 ± 1.73	5.50 ± 0.81	6.16 ± 0.43
${h}_{4}$ (km)	10	100	Infinity	69.58 ± 13.87	70.05 ± 3.50	65.68 ± 1.96
Fitness (RMS)				0.0229	0.02245	0.02242

Nevertheless, Rai et al.^50^ examined the teleseismic data from 17 broadband seismometers along a 700 km long profile and discovered a Moho depth of about 75 km above the South Karakorum Fault in Ladakh, India, which is relatively near to the vPSOGWO inverted crustal thickness.

#### Example 5: Joint inversion of DC and MT data over Broken Hill, Australia

**Figure 17:**
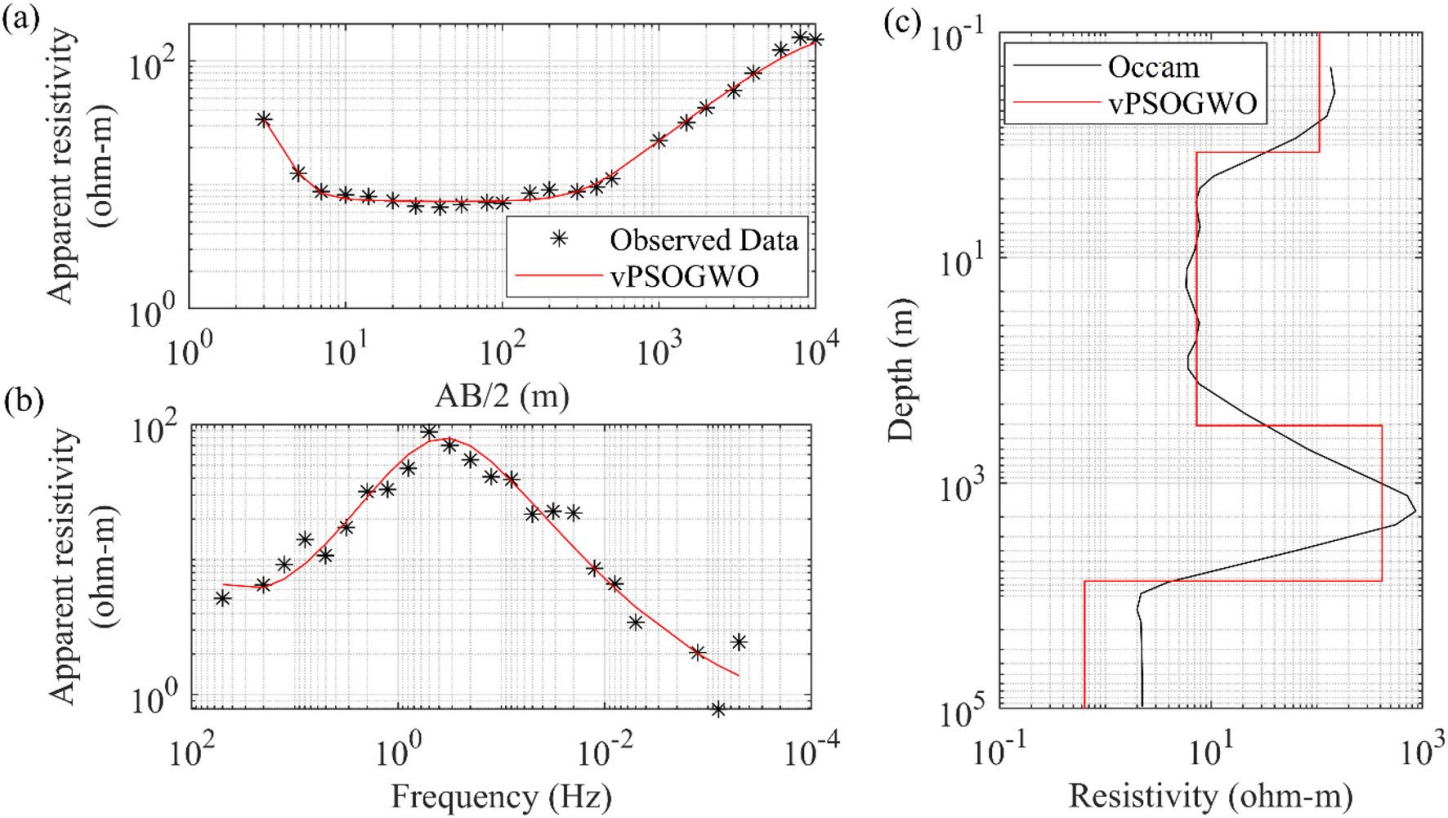
Four-layers DC and MT field data near Broken Hill in South Central Australia for joint inversion: (**a**) DC observed (*) and the best-fitted apparent resistivity curves, (**b**) MT observed (*) and the best-fitted apparent resistivity curves, (**c**) 1D mean model (bpdf > 68.27% CI) inverted by vPSOGWO scheme (red).

To understand the versatility of the proposed algorithm, an example of joint inversion of DC and MT datasets was carried out. There are inherent uncertainties in any geophysical measurement. It is advised to utilize an integrated strategy due to the ambiguities in geophysical methodologies^51,52^. It is also possible that ambiguities may be reduced with extensive and diverse datasets, leading to a more reliable and accurate model. The processing can be sped up, and the interpretation is made simpler using various datasets to find the same physical attributes. For instance, the electrical resistivity of the earth's subsurface, which is influenced by temperature and permeability^53^, is a shared physical attribute between the DC and MT nonlinear datasets. Both DC and MT methods can produce a model of the subsurface relating variations in the resistivity to variations in lithology. Although both strategies are inherently ambiguous, their combined interpretation is more rational and produces superior outcomes^54,55^. These techniques provide more consistent and trustworthy subsurface models and variations than those derived by individual inversions^33,56^. The DC and MT datasets were obtained in the period range of 0.02 to 1995s from a site near Broken Hill in South Central Australia, where Schlumberger sounding data was received over a spreading of 20 km^57,58^. Using the novel vPSOGWO technique, the apparent resistivity data was inverted here. The inverted findings were seen together with the search range taken in this investigation, as given in Table 11. Figure 17a displays the measured and computed apparent resistivity curves. In contrast, Fig. 17b shows the 1D depth model derived by the vPSOGWO with an RMS error of 0.09486, comparable with the result inverted using Occam's inversion^58^.

**Table 11:** Mean model with the level of uncertainty in the posterior utilizing vPSOGWO joint inversion of DC and MT data near Broken Hill in South Central Australia.

Layer parameters	Search range		Joint inverted value
	Lower	Upper	vPSOGWO (PDF > 68.27% CI)
${\rho }_{1}$ (Ωm)	40	500	105.58 ± 4.90
${\rho }_{2}$ (Ωm)	1	10	7.33 ± 0.02
${\rho }_{3}$ (Ωm)	100	1000	416.55 ± 11.28
${\rho }_{4}$ (Ωm)	0.1	5	0.64 ± 0.01
${h}_{1}$ (m)	1	10	1.16 ± 0.02
${h}_{2}$ (m)	50	500	307.73 ± 1.55
${h}_{3}$ (m)	1000	10,000	7122.62 ± 14.68
Fitness (RMS)			0.0949

#### Example 6: Joint inversion of DC and MT data over Central Puga Valley, India

**Figure 18:**
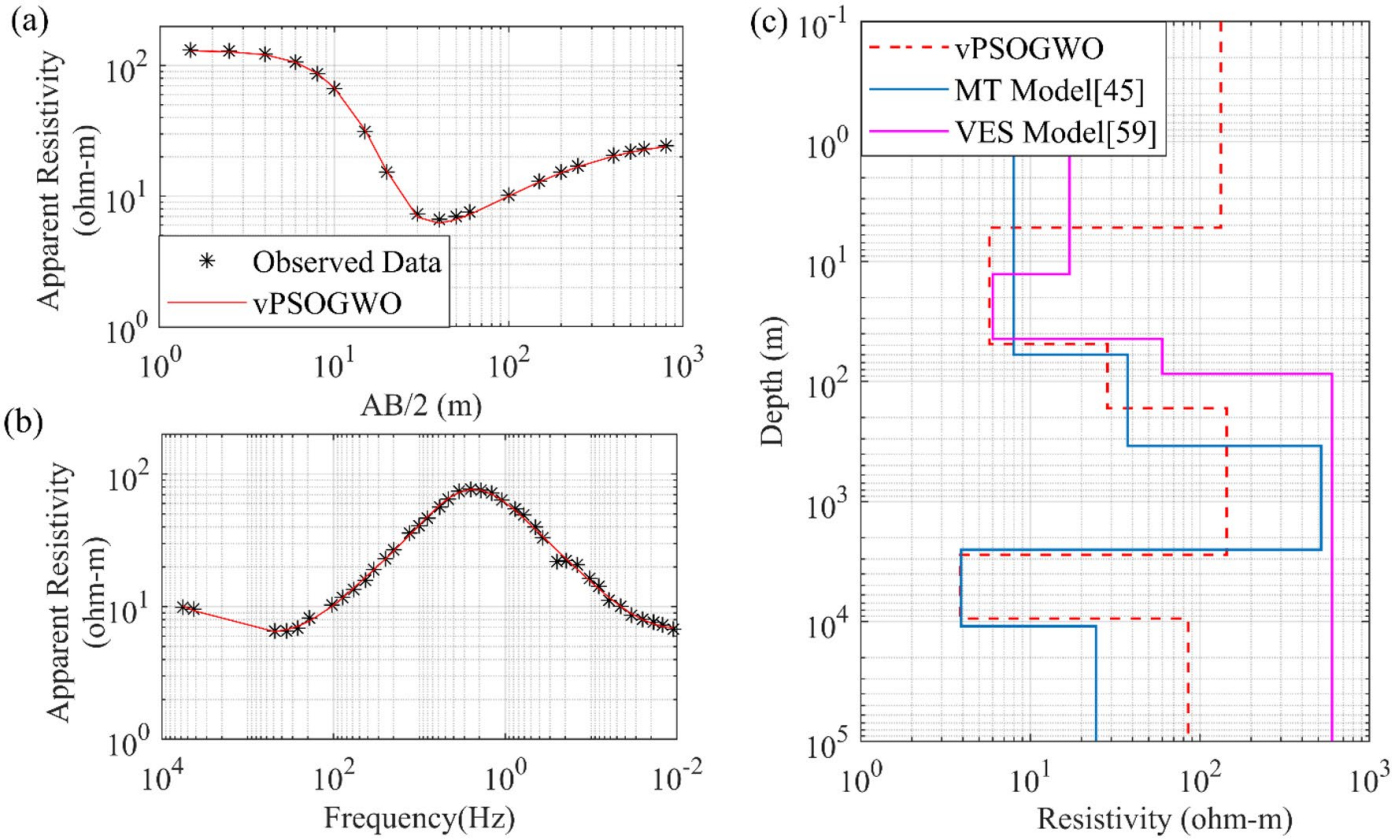
The inverted response by joint inversion based PSO, GWO, and vPSOGWO with a valid model (black) over Central Puga Valley, India: (**a**) DC observed and best-fitted apparent resistivity curves, (**b**) MT observed and best-fitted apparent resistivity curves and (**c**) 1D mean model (bpdf > 68.27% CI) inverted by vPSOGWO scheme (red).

The geothermal central Puga valley of Ladakh was used as another field example for the 1D joint inversion of DC and MT resistivity sounding data based on vPSOGWO technology, and the outcome is analysed. With search range, inverted mean model and posterior uncertainty based on recent joint inversion is shown in Table 12. In earlier published research, a variety of techniques were used to interpret the MT data using constraints obtained from the original model^45^, and the ANN-based Levenberg–Marquardt algorithm was utilized to interpret the VES data using inversion of specific data that was unable to generate reliable models. These aforementioned interpretations were either able to determine the shallow or deeper zone^59^. Figure 18c shows a conductive zone with a thickness of 43.58 ± 0.09 m, which is quite similar to the thickness of the shallow conductive geothermal layer established by drilling information^60^ and better than the result of published individual model^45,59^. Also as shown in Fig. 18 and Table 12 below the depth of around 2778 m has another conductive zone of exceptionally low resistivity is 3.90 ± 0.01 Ωm with 6680.55 ± 63.22 m thickness in the inverted model, which is comparable to the model provided by Harinarayana et al.^45^. Through this examination, a more precise identification of the shallow and deeper geothermal zones was made, which is necessary for precisely estimating of the geothermal reservoir.

**Table 12:** Mean model with the level of uncertainty in the posterior utilizing vPSOGWO joint inversion of DC and MT data over Central Puga Valley, India.

Layer parameter	Search range		Joint inverted value
	Lower	Upper	vPSOGWO (PDF > 68.27% CI)
*ρ*_1_ (Ωm)	80	200	133.27 ± 0.83
*ρ*_2_ (Ωm)	0.1	10	5.74 ± 0.08
*ρ*_3_ (Ωm)	20	100	28.49 ± 0.36
*ρ*_4_ (Ωm)	80	1000	143.73 ± 2.18
*ρ*_5_ (Ωm)	0.1	10	3.87 ± 0.17
*ρ*_6_ (Ωm)	10	500	85.28 ± 0.62
h_1_ (m)	0.1	10	5.22 ± 0.02
h_2_ (m)	5	50	43.57 ± 1.34
h_3_ (m)	50	700	119.04 ± 12.38
h_4_ (m)	1000	5000	2610.70 ± 45.75
h_5_ (m)	1000	10,000	6680.55 ± 63.22
Fitness (RMS)			0.014

## Conclusions

In the present paper, we use nonlinear DC and MT-sounding synthetic data distorted with 10% Gaussian noise to demonstrate the applicability and novelty of the new strategy vPSOGWO technique in handling multi-layered resistivity parameters and nonlinear individual and joint inversion issues. Finally, we applied the vPSOGWO approach to field data over various geological setups, including Digha, India; Sundar Pahari, India; Puga Valley of Ladakh, India; Chatham, New Brunswick, Canada; and Broken Hill in South Central Australia. The aforementioned datasets were inverted with 10 population sizes and 1000 iterations, which is executed 1000 times producing several workable models. Further calculations include the posterior Bayesian Probability Density Function with 68.27% CI for estimating the global mean model and its uncertainty. The estimated models are more accurate and relatively consistent than those derived from earlier techniques. Also, vPSOGWO can avoid the issue of premature convergence that GWO and PSO encounter, particularly by balancing the features of exploration and exploitation, trapping at local minima, and lowering posterior uncertainty. It was established that the suggested current approach is equivalent to or better than the findings of the GWO and PSO and corresponds well with the present data to validate. If the current technique is investigated further using the aforementioned novel high-dimension individual and joint inversion algorithms, the detail volume of the earth's subsurface structure can be accurately estimated, even in the complex geological environments, and its actual potential for various geophysical applications can be offered.

## Data Availability

The data that support the findings of this study are available from the corresponding author upon reasonable request.
